# L-Arginine-Nitric Oxide-Asymmetric Dimethylarginine Pathway and the Coronary Circulation: Translation of Basic Science Results to Clinical Practice

**DOI:** 10.3389/fphar.2020.569914

**Published:** 2020-09-29

**Authors:** Attila Cziráki, Zsófia Lenkey, Endre Sulyok, István Szokodi, Akos Koller

**Affiliations:** ^1^ Medical School, Heart Institute, University of Pécs, Pécs, Hungary; ^2^ Szentágothai Research Centre, University of Pécs, Pécs, Hungary; ^3^ Institute of Public Health and Health Promotion, University of Pécs, Pécs, Hungary; ^4^ Department of Morphology and Physiology, Faculty of Health Sciences, Semmelweis University, Budapest, Hungary; ^5^ Research Center for Sports Physiology, University of Physical Education, Budapest, Hungary; ^6^ Department of Physiology, New York Medical College, Valhalla, NY, United States

**Keywords:** coronary artery disease, nitric oxide (NO), L-arginine, asymmetric dimethylarginine (ADMA), ischemia/reperfusion, coronary revascularization, pericardial fluid

## Abstract

By 1980, it was thought that we already knew most of the major mechanisms regulating vascular tone. However, after the somewhat serendipity discovery that endothelium is involved in mediation of relaxation to acetylcholine, a whole new world opened up and we had to rewrite our concept regarding vascular function and its regulation (not to mention many other fields). The new player was an endothelium derived relaxing factor, which molecular constitution has been identified to be nitric oxide (NO). This review summarizes the major molecular steps concerning how NO is synthetized from L-arginine. Also, the fate of L-arginine is described *via* the arginase and methylation pathways; both of them are affecting substantially the level and efficacy of NO. *In vitro* and *in vivo* effects of L-arginine are summarized and controversial clinical findings are discussed. On the basis of the use of methylated L-arginines, the vasomotor effects of endothelial NO released to agonists and increases in flow/wall shear stress (a major biological stimulus) is summarized. In this review the role of NO in the regulation of coronary vascular resistance, hence blood flow, is delineated and the somewhat questionable clinical use of NO donors is discussed. We made an attempt to summarize the biosynthesis, role, and molecular mechanisms of endogenously produced methylated L-arginine, asymmetric dimethylarginine (ADMA) in modulating vascular resistance, affecting the function of the heart. Additionally, the relationship between ADMA level and various cardiovascular diseases is described, such as atherosclerosis, coronary artery disease (CAD), ischemia/reperfusion injuries, and different types of coronary revascularization. A novel aspect of coronary vasomotor regulation is identified in which the pericardial fluid ADMA and endothelin play putative roles. Finally, some of the open possibilities for future research on L-arginine-NO-ADMA signaling are highlighted.

## Introduction

After the original observation by Furchgott and Zawadzki (Furchgott and Zawadzki, 1980) it took more than a decade to identify the chemical nature and biochemical pathway of endothelium-derived relaxing factor (RF: “Robert Furchgott”) as it was called in the original paper. The factor mediating the vasomotor effect of acetylcholine (ACh) turned out to be a gas molecule, nitric oxide (NO), which—among many other biological effects—in a very potent manner relaxes vascular smooth muscle (Loscalzo, 2013). It was revealed that L-arginine (L-Arg) is the substrate for nitric oxide synthases (NOS) [endothelial (e), neuronal (n), and inducible (i)], which then produces NO and L-citrulline (Moncada and Higgs, 2006). L-Arg, however, can be used by arginase and also L-Arg methylated, thus both pathways can decrease its availability for NOS. Interestingly, the chemical nature of NO was assumed primarily on the basis of the vasomotor responses could be inhibited by methylated L-arginines (Fukuto et al., 1990; Ji et al., 2009). The biological importance of NO was further substantiated by isolated organ (Koller et al., 1994) and animal (Trochu et al., 2003) studies showing that NO is involved in the control of vascular resistance, blood flow, and systemic blood pressure (Rees et al., 1996).

**Figure 1:**
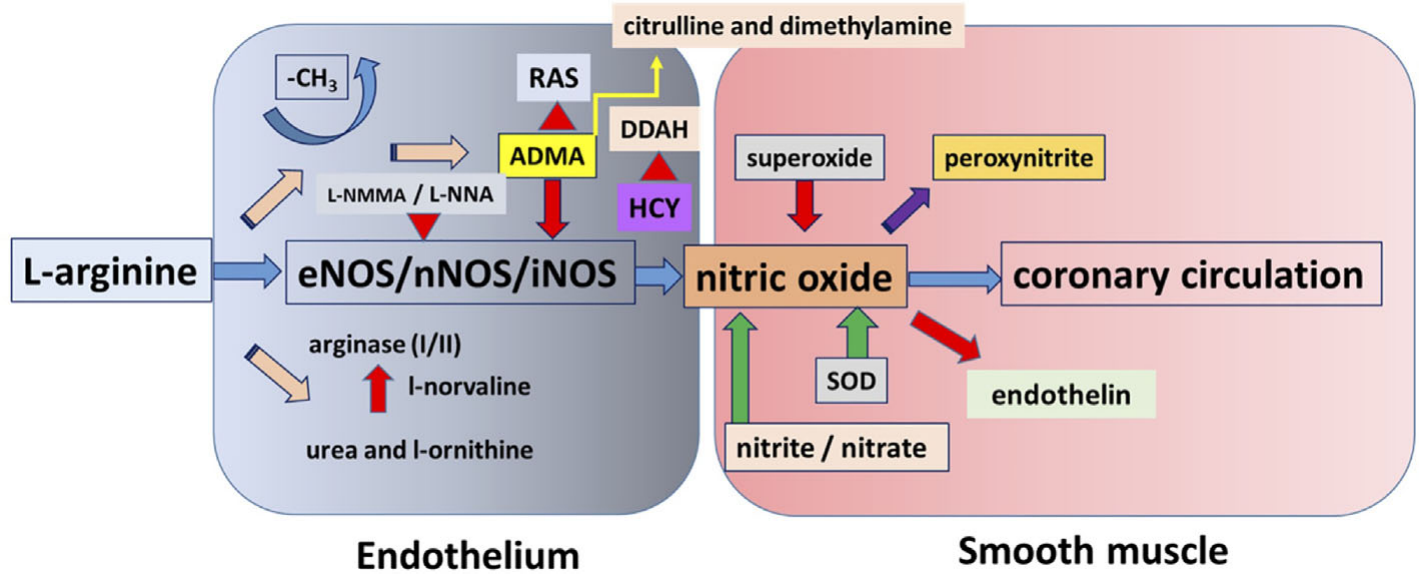
This figure shows a simplified overview of nitric oxide (NO) pathway with most of the molecular steps. The substrate for nitric oxide synthases, the amino-acid L-arginine can enter into three main metabolic pathways. It is either used by arginase, producing urea, and L-ornithine or used by nitric oxide synthase (NOS) to produce NO or methylated, producing methylated L-arginines, which being a false substrate inhibit NOS. Other metabolic pathways are connected that are modifying the level of NO or affected by NO.

After the discovery of the steps of the early signaling of the NO pathway, they were investigated on the cardiovascular system in order to be used in clinical practice. NO has multiple biological roles/activities, but it is a short-lived molecule, thus many investigations were done aiming to elucidate the possibility of improvement of cardiovascular function by administering activators and inhibitors of the early part of the NO-signaling pathway (**Figure 1**). Thus, in this review, we aimed to summarize some of the main findings, conflicting results, controversies, significant development, and the potential clinical use of the early part of NO signaling, focusing on their vasomotor effects, primarily in the coronary circulation (**Figure 1**).

### L-Arginine Used by the Arginase Pathway

L-Arg is metabolized to urea and l-ornithine by the arginase enzyme *via* hydrolysis in the urea cycle. The two arginase isoforms (arginase I and arginase II) are established to have different tissue as well as cellular distribution and immunoreactivity. These isoforms are both present in the endothelium of the vascular bed and they have been identified to have an important role in NO synthesis, thus in maintaining proper endothelial function. L-Arg is the common substrate for both NOS and arginase. The upregulation of arginase decreases L-Arg bioavailability and consequently decreases NO synthesis (Jenkinson et al., 1996; Bachetti et al., 2004). Furthermore, arginase activity can result in NOS uncoupling and the production of superoxide and peroxynitrite. These compounds can also weaken NOS function (Durante et al., 2007). The co-expression of arginase and NOS in the vascular endothelial and smooth muscle cells (SMC) also supports the interplay between these enzymes in the L-Arg–NO pathway (Hein et al., 2003). Moreover, reactive oxygen species and pro-inflammatory cytokines can increase the activity of arginine methyltransferases, inhibit dimethylarginine dimethylaminohydrolase (DDAH), and up-regulate arginase. These effects result in the increased synthesis of methylarginines and ornithine (Hein et al., 2003). It is worth mentioning that peroxynitrite enhances arginase activity which then increases peroxynitrite production leading to the worsening of vascular dysfunction (Sankaralingam et al., 2010).

Arginase can also lead to impaired vascular function *via* a NO-independent way. Specifically, l-ornithine can be further metabolized either to polyamine or to proline which may induce vascular SMC growth and proliferation, and collagen deposition, respectively. These mechanisms may proceed to neointima formation and remodeling of the vascular wall (Li et al., 2001). It has been established that both the expression and the activity of arginase are increased in coronary arteries from ischemic myocardium and arginase inhibition mediates cardioprotection in ischemia-reperfusion injury (Shemyakin et al., 2012; Kovamees et al., 2014). Kuo et al. showed that increased arginase activity can also contribute to the inhibition of flow-induced dilation of coronary vessels (Kuo and Hein, 2013), and also to the development of hypertension as well as aging (Durante et al., 2007).

### Vasomotor Role of L-Arginine: Potential Clinical Use

L-Arg has many biological functions—among others—being the substrate for NO-synthases producing NO, which plays important roles in maintaining endothelial health by promoting vasodilatation, inhibiting endothelial cell apoptosis, leucocyte adhesion, and thrombocyte aggregation (Cocks et al., 1985; Palmer et al., 1988a; Palmer et al., 1988b). Beside these actions, it also exerts its beneficial effect on microcirculation by smooth muscle relaxation, inhibition of leucocyte adhesion, thrombocyte aggregation, and the expression of adhesion molecules as well as chemotactic peptides through a NO-dependent pathway. NO increases the intracellular synthesis of cyclic GMP (cGMP) in smooth muscle cells *via* the activation of guanylate cyclase. In large vessels cGMP mediates most of the biological effects of NO (Tousoulis et al., 2007). In contrast, flow/shear stress-induced release of NO seems to effect calcium sensitivity of smooth muscle in microvessels, which then leads to dilation (Ungvari et al., 2001). Experiments were carried out on isolated arterioles of rat gracilis muscle. Measurement of endothelial calcium concentration was assessed with a fluorescence method. The main finding of the study was that NO-mediated vasodilatation in response to shear stress results in only a mild increase in the endothelial calcium concentration. The authors concluded that in healthy, intact endothelium of skeletal muscle arterioles, shear stress does not stimulate calcium from intracellular stores and induces only a minor influx of extracellular calcium (Ungvari et al., 2001). Thus, shear stress induced arteriolar dilatation seems to be a calcium-independent signaling pathway in arteriolar endothelial cells.

**Figure 2:**
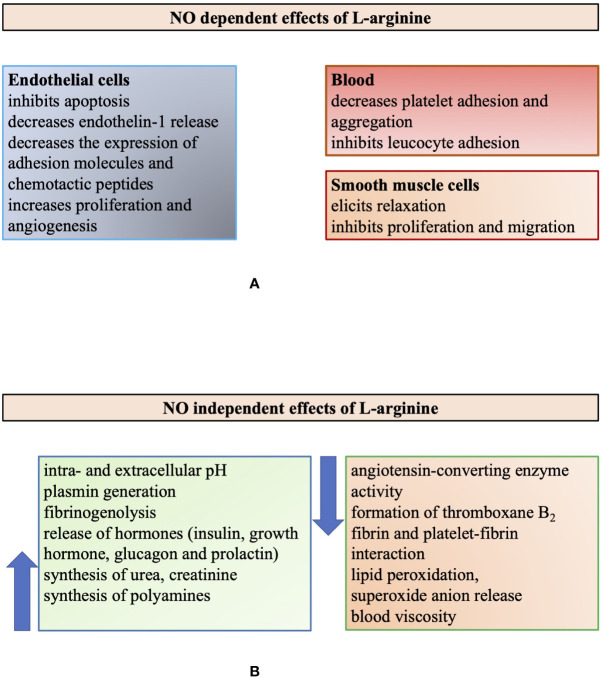
Illustrating the multiple biological roles of L-arginine modified after Tousoulis et al., 2002. Summarizing the multiple biological actions of L-arginine, which are either nitric oxide (NO) dependent (panel **A**) or independent (panel **B**).

It is of note, however, that L-Arg has many biological actions that are not related to NO (Tousoulis et al., 2002). L-Arg is important in the regulation of both intra- and extracellular pH and has an important role in reducing lipid peroxidation and the elimination of superoxide anion (**Figure 2**). High concentration of L-Arg can decrease blood viscosity by inhibiting the binding of macromolecules to the surface of red blood cells *via* insulin release (Walter et al., 2000). The authors assumed that this decreased blood viscosity may explain the improved blood flow after L-Arg administration seen in previous studies (Bode-Boger et al., 1994; Quyyumi, 1998). L-Arg regulates blood pressure by directly inhibiting angiotensin-converting enzyme (ACE) (Higashi et al., 1995). It affects fibrinolysis *via* this inhibition and the decrease of thromboxane B2 synthesis, an increase of plasmin generation and fibrinogenolysis (Udvardy et al., 1997). It can also induce the release of insulin that can result in further vasodilatory effect and reduced platelet aggregation.

These are important facts to keep in mind, in order to understand and interpret the complex effects of L-Arg administration (supplementation) to biological systems and in humans. The complexity is highlighted in **Figure 2**, summarizing the NO-dependent and independent action of L-Arg (Tousoulis et al., 2002).

L-Arg has a beneficial effect on endothelial function in patients with ischemic heart disease and dilates the atherosclerotic, stenotic coronary arteries (Tousoulis et al., 1999; Tousoulis et al., 2007). In humans normal daily diet contains 5.4 g L-Arg from which approximately 30–50% enters the circulation (Tangphao et al., 1999). The serum level of this amino acid is not constant throughout the day and depends partially on the exogenous intake of L-Arg finally resulting in a plasma concentration of 95–250 μmol/l under physiological conditions (Tangphao et al., 1999). The concentration of this substance in the endothelial cells ranges from 1 to 2 mmol/l (Tousoulis et al., 2002).

It is worth mentioning, however, that the methylated derivatives of arginine act as inhibitors of NOS *via* competing with arginine for the active site of NOS. The most frequently used inhibitors in experimental studies are: N^G^-monomethyl-L-arginine (L-NMMA), asymmetric dimethylarginine (ADMA), N^G^-nitro-L-arginine (L-NNA), and N^G^-nitro-L-arginine methyl ester (L-NAME).

It is still not clear whether sustained administration of L-Arg has any effect on clinical endpoints in patients with atherosclerotic coronary artery disease (CAD). Inhibition of arginase seemed to increase intracellular L-Arg bioavailability better than done by exogenous L-Arg supplementation (Berkowitz et al., 2003).

### L-Arginine: Studies From Cultured Cells to Organism

We have made a search in literature regarding the vasomotor and hemodynamic effects of L-Arg (Khalaf et al., 2019). There have been many interesting studies and findings and we include here those showing various results under various conditions to highlight the complexity of issues.

#### Cultured Endothelial Cells

In 1988, Palmer and his working group demonstrated that NO can be synthesized by porcine aortic endothelial cells when cultured with L-Arg as measured through the release of NO with bioassay, chemiluminescence, and mass spectrometry (Palmer et al., 1988a). They found that the increase in the amount of NO is reversible after the administration of L-Arg, but not D-arginine, and that NO was synthesized from the terminal guanidino nitrogen atoms of L-Arg. In 1990, Gold et al. proved that endogenous L-Arg concentration is an important factor in the regulation of cGMP synthesis (Gold et al., 1990). They observed a significant decline of cGMP level in case the concentration of L-Arg decreased in endothelium-intact pulmonary artery rings.

#### L-Arginine Relaxes Smooth Muscle of Isolated Large Arteries and Dilates Arterioles

As the substrate of endothelial NOS (eNOS), the effects of L-Arg are mediated mainly *via* the increase of NO production (Palmer et al., 1988a; Palmer et al., 1988b). It could then reasonably be assumed that exogenous L-Arg will elicit dilation of the vessels. Indeed, in isolated skeletal muscle arterioles (~100 micron in diameter) L-Arg (10^−5^ to 10^−3^ M) evoked dose dependent dilations, similarly to that of ACh (Sun et al., 1992). Administration of the NOS-inhibitor L-NNA inhibited both the ACh and the L-Arg induced vasodilatation of the arterioles. Removal of the endothelium also resulted in diminished Ach and the L-Arg induced vasodilatation. Based on these results, the authors concluded that in skeletal muscle arterioles the dilations to ACh and L-Arg are endothelium dependent. Kaley et al. (1992) demonstrated the role of the L-Arg pathway in NO biosynthesis when examining the effect of specific NOS-inhibitors and L-Arg. They measured the change in arteriolar diameter with *in vivo* television microscopy. They found that the ACh-induced vasodilatation of the arterioles was inhibited in the presence of L-NMMA or L-NNA, but this effect was reversed by administration of 1 mM L-Arg.

#### Overestimation of the Role of Nitric Oxide

We have to note, that it is very difficult to measure NO, and most of the time effects of methylated L-arginines (MLA) were taken as equal to NO mediation. Our studies, however, showed that higher doses of L-NNA, such as 10^−4^ M, or 10^−3^M (used frequently in many studies), inhibited not only NO related diameter responses but also those associated with prostaglandins, and even adenosine (Koller et al., 1993). Thus it is likely that the mediator role of NO was overestimated in many previous studies using high concentrations of MLAs.

#### Effects of L-Arginine Administration on the Function of Isolated Heart

The working group of Agullo (Agullo et al., 1999) investigated the effect of L-Arg when applied in the rat heart model of ischemia-reperfusion injury. They found that the addition of 3 mmol/l arginine to the perfusate of isolated rat hearts during the experiment increased the release of cGMP and prevented myocardial cell death. The authors concluded that the beneficial effect of L-Arg supplementation is predominantly mediated by the increased cGMP availability during reperfusion.

Fujita and coworkers examined the effect of 3-week orally administered L-Arg in spontaneously hypertensive rats (SHR) (Fujita et al., 2000). Systolic blood pressure was measured once a week. They found that blood pressure did not change. After the treatment the heart was excised and perfused with Krebs-Henselite solution in a Langendorff preparation. Perfusion flow was measured with a drop counter. After stabilization of the solution, adenosine was used to achieve maximal vasodilatation of the coronary arteries. After the infusion of 10^−4^ M L-NMMA, the change in coronary perfusion flow was calculated by percentage. The myocardium was weighed after the removal of the atria and aorta and there was no change in cardiac hypertrophy. The basal coronary perfusion resistance (CPR) was not changed in SHR treated with L-Arg for 3 weeks. However, the L-NMMA induced CPR response was more prominent in the treatment group of SHR when compared to the control SHR group. The authors concluded that coronary flow reserve was impaired in SHR, but L-Arg restored it.

#### Human and Experimental Animal Studies With L-Arginine

L-arginine supplementation has been identified to improve endothelial function both in animal and human subjects (El-Kirsh et al., 2011; Morita et al., 2014). Experimental studies have shown that L-Arg supplementation might be beneficial after coronary angioplasty (Tousoulis et al., 2002), resulting from decreased vascular remodeling and neointimal thickening caused by the perivascular delivery of this amino acid (Bosmans et al., 1999). Dysfunction of the L-Arg-eNOS pathway is involved in the pathogenesis of CAD (Tousoulis et al., 2002). It has also been demonstrated that L-Arg may play a role in both the prevention and progression of CAD (Tousoulis et al., 2002). There are several human and experimental studies investigating the effect of L-Arg treatment on vascular health, taking into account that this amino acid is the substrate for NO-synthase, thus NO production, which is essential in maintaining proper endothelial function.

In 1992, Hishikawa et al. (1992) demonstrated the blood pressure lowering (mean arterial pressure decreased from 79.3 ± 3.9 to 68.8 ± 2.2 mmHg) and heart rate increasing (approximately 8% increase in heart rate) effect of intravenous L-Arg in normotensive patients. The urine concentration of nitrite/nitrate increased significantly by 142.1 ± 12.4% after L-Arg administration compared to the baseline value, and the plasma concentration of cGMP also increased.

Cooke et al. were the first to show that in hypercholesterolemic rabbits, L-Arg supplementation for 10 weeks improved endothelial-dependent vasodilatation and reduced both the intimal thickness and the area of atherosclerotic lesion of the thoracic aorta (Cooke et al., 1992). The ACh-induced maximal relaxation of the thoracic aortic ring was considerably less in the hypercholesterolemic group compared to the control group and the group that was supplemented with L-arginine-HCl in drinking water (61 ± 5 *vs*. 89 ± 2% *vs*. 73 ± 3, for the hypercholesterolemic *vs*. control *vs*. supplemented group respectively; p < 0.05). After staining the intimal lesion of the surface area, the authors found that there was no lesion in case of control animals, in hypercholesterolemic rabbits this area was about 40%. The area that was affected by intimal lipid accumulation proved to be less than 10% in hypercholesterolemic rabbits supplemented with L-Arg. The reduction of intimal thickness was more pronounced in the distal segment of the vessel resulting in 86% reduction in hypercholesterolemic rabbits supplemented with L-Arg.

Dubois-Rande et al. (1992) examined thirteen hypercholesterolemic patients with diffuse non-stenotic coronary atherosclerosis of the left anterior descending coronary artery (LAD) as proven by coronary angiography. LAD diameters and coronary blood flow (CBF) were determined with Doppler flow velocity measurements. Intracoronary infusion of ACh infusion induced a dose-dependent decrease of the diameter of the distal LAD, which response was enhanced by the intracoronary administration of L-Arg.

Egashira et al. (1996a) also examined the effect of the intracoronary infusion of L-Arg on the vasodilatation of coronary arteries in patients with angina pectoris but no sign of coronary atherosclerosis during coronary angiography (i.e., patients with microvascular angina pectoris). The study protocol was the following: 30 min after completion of the diagnostic angiography ACh was administered intracoronary through the guiding catheter. Ten minutes later, intracoronary infusion of L-Arg was performed and simultaneously the ACh study was repeated. Another 10 min after the completion of the L-Arg infusion, isosorbide dinitrate was infused *via* the intracoronary catheter and in the last step, papaverine was infused in the same way. The diameter at the proximal segment of the LAD was measured and CBF was detected with a Doppler flow velocity catheter. The ACh infusion increased blood flow more in control subjects than in patients with microvascular angina pectoris, which was significantly attenuated with L-arginine infusion in patients but not in control subjects. The latter effect of L-Arg was also confirmed by Quyyumi and coworkers in a study involving 32 patients with coronary atherosclerosis or its risk factors (Quyyumi et al., 1997a).

Bode-Böger et al. reported that a 30-min intravenous infusion of 30 g L-Arg resulted in a decrease of systolic blood pressure (−4.3 mmHg; p < 0.05) and in a more prominent drop in diastolic blood pressure (−6.7 mmHg; p <0.001) when compared to placebo in healthy human volunteers (Bode-Boger et al., 1994). They also detected that the urinary excretion of cGMP—the target of NO—increased by 65.4%, and the nitrate by 79.7% after L-Arg treatment. In addition, the intracellular concentration of cGMP inside platelets was increased by 43% and thrombocyte aggregation was inhibited by 32.7% after L-Arg administration.

Later, Böger and coworkers examined the effect of a 16-week dietary L-Arg intake in hypercholesterolemic rabbits (Boger et al., 1997). In this experimental model of hypercholesterolemia the serum level of ADMA (eNOS inhibitor) is elevated. The supplementation of L-Arg restored endogenous NO production and improved endothelial-dependent relaxation of the thoracic aorta potentially by increasing the L-Arg/ADMA ratio. The authors concluded that the L-Arg/ADMA ratio may be important in the regulation of NO synthesis in hypercholesterolemia by providing a higher level of L-Arg.

In an important clinical study, Tousoulis et al. investigated the effect of the intracoronary infusion of L-Arg in patients with known CAD and stable angina as well as in subjects with normal coronary angiograms (Tousoulis et al., 1999). They found that L-Arg administration resulted in a marked vasodilatation of both normal and diseased coronary arteries. The mean percentage change of luminal diameter from baseline was 8.5, 6.7, and 11.7% for the proximal, distal, and stenotic segments of coronary arteries in patients with ischemic heart disease, respectively. In the control group these changes were 10.4 and 6.5% for the proximal and distal segments of coronary arteries, respectively.

#### Oral Supplementation With L-Arginine

Obviously, intravenous or intra-arterial administration of L-Arg are inconvenient ways for patients, thus studies were necessary to examine the effect of oral supplementation of L-Arg in humans. Based on the beneficial results of L-Arg supplementation observed in hypercholesterolemic rabbits, Clarkson et al. confirmed the favorable effect of 3x7 g daily intake of this amino acid for 4 weeks on the endothelium-dependent vasodilatation in young patients with hypercholesterolemia (Clarkson et al., 1996). They measured the diameter of the brachial artery at rest, in response to reactive hyperemia, at rest afterwards and finally after the administration of sublingual nitroglycerin. They observed a significant improvement of the post-occlusion endothelium-dependent vasodilatation of the brachial artery from 1.7 ± 1.3 to 5.6 ± 3.0% (p < 0.001) in hypercholesterolemic subjects, while taking L-Arg compared to the placebo group (2.3 ± 1.9 to 2.3 ± 2.4%; p=NS) there was a considerable increase in diameter in the treated compared to placebo group (3.9 ± 3.0% *vs*. 0.1 ± 2.2% in the L-Arg *vs*. placebo group, respectively; p < 0.001). Lerman at al. investigated the effect of chronic (6 months) administration of 3 g L-Arg daily in 26 patients without significant CAD (Lerman et al., 1998). CBF reserve was investigated with the infusion of ACh selectively into the LAD. They noticed that the oral supplementation of L-Arg improved ACh-induced increase in CBF (149 ± 20 *versus* 6 ± 9%, p< 0.05) and significantly improved the symptoms (i.e., chest pain) of the patients. In addition, they also found that L-Arg decreased the plasma endothelin concentration (by 35 ± 5%) and concluded that it can be partially the reason why L-Arg supplementation improves endothelial function. These findings show an important link between NO and endothelin pathways. Luscher et al. previously proved that L-Arg as a substrate of NO also decreases endothelin-I (ET-I) release, because NO can reduce ET-I production in endothelial cells (Luscher et al., 1990) and can regulate the effect of ET-I on the endothelin-B receptor (Di Luozzo et al., 2000).

Importantly, not every human study showed beneficial effects of L-Arg supplementation. For example, Blum et al. (2000) found that a 1-month daily oral treatment of 9 g L-arginine did not have any effect on NO levels measured in serum by a chemiluminescent technique (19.3 ± 7.9 *versus* 18.6 ± 6.7 μmol/l, p=0.546) and flow-mediated dilatation of the brachial artery (11.9 ± 6.3 *versus* 11.4 ± 7.9%, p=0.742). Similarly, Walker et al. reported that 2-week supplementation of daily 3x5 g oral L-Arg did not improve endothelial-dependent vasodilatation in patients with CAD as measured by the blood flow in both forearms using venous occlusion plethysmography (Walker et al., 2001). First, they found a significant improvement in ACh-induced vasodilatation after the intra-arterial administration of L-Arg that remained practically unchanged after the 2 weeks of oral supplementation of the amino acid (18.3 ± 1.8% *vs*. 26 ± 9.8% increase in blood flow compared with baseline after the intra-arterial and oral administration of L-Arg, respectively; p=NS), although the high standard error of the mean may indicate insufficient number of data.

In an interesting study of Neri et al. (2010), 80 pregnant women with mild chronic hypertension were enrolled. In this placebo-controlled trial, oral L-Arg 2 g twice a day was administered. After 10–12 weeks, the BP change as the primary outcome of the study was examined by a 24-h ambulatory BP monitoring. There was no significant difference in BP changes between the placebo and the treatment group. However, L-Arg supplementation was associated with less need of antihypertensive drugs and fewer maternal and neonatal complications.

Exercise has been shown to increase eNOS activity, and superoxide dismutase (SOD) expression, both of them resulting in increased release and bioavailability of endothelium-derived NO with the consequent increase in vascular diameter and reduction of peripheral resistance (Koller et al., 1995; Dornyei et al., 2000).

Based on similar logic, Ceremuzynski et al. investigated the effect of oral 6 g/day L-Arg for three consecutive days in patients with CAD and stable angina pectoris (Ceremuzynski et al., 1997). In their study, treadmill exercise test according to the modified Bruce protocol was performed before and after L-Arg treatment. Exercise tolerance and the mean exercise time to maximal ST-segment depression increased (approximately 14% improvement in exercise tolerance and 24% increase in the mean exercise time to maximal ST-segment depression), while ST-depression was reduced in the treatment group (−6.8 ± 2 *vs*. −5.5 ± 2 mm, p < 0.05) when compared to placebo.

Because the methods used for L-Arg supplementations varied, in 2011 a meta-analysis of 11 randomized, double-blinded, placebo-controlled trials examined the effect of a median daily dose of 9 g arginine (ranging from 4 to 24 g daily) administered orally for a median of 4 weeks (Dong et al., 2011). L-Arg supplementation caused a 5.39 mmHg drop in systolic blood pressure (95% CI −8.54 to −2.25, p = 0.001) and lowered diastolic blood pressure by 2.66 mmHg (95% CI −3.77 to −1.54, p < 0.001). Although the findings of this meta-analysis support the beneficial effects of L-Arg administration on hemodynamic parameters, it should be emphasized that comorbidities of the patients, baseline blood pressure, dosage of L-Arg supplementation, and the duration of therapy differed considerably between the trials.

There are conflicting results regarding the outcome of oral L-Arg supplementation in clinical studies, which can be due to the heterogeneous patient population and the treatment protocol used in investigating the effect of this amino acid. Although the maximal effective dose of L-Arg supplementation with only minor gastrointestinal side effects seems to be 21 g, daily divided into three doses (Clarkson et al., 1996), there is a need for larger prospective clinical trials to come to a conclusion regarding the efficacy of dietary L-Arg intake in the prevention and treatment of cardiovascular diseases (CVD).

Explaining the somewhat conflicting results, Boger vs. Böger came up with the term “L-arginine paradox” (Boger, 2004) meaning that additional dietary intake of this amino acid can have a favorable effect on vasodilatation and other hemodynamic parameters despite the substantial amount of available L-Arg for eNOS. However, it is worth mentioning—as discussed above—that L-Arg is the substrate for both NO and for methylated arginine products, which may outweigh the beneficial effects of L-Arg on NO synthesis. Schneider et al. (2015) investigated the effect of a long-term (i.e., 3 and 6 months) oral supplementation of 10 g/day L-Arg in a placebo-controlled trial of patients with peripheral artery disease and patients with CAD. This dose is twice the average daily intake of this amino acid in a normal Western diet. They concluded that the main effect of oral L-Arg supplementation is enhancing the bioactivity and not the synthesis of NO induced by eNOS potentially by preventing the renal loss of nitrite. According to their study, the dietary intake of L-Arg should not be less than 10 g a day in order to see the beneficial effects of the amino acid. Lundberg and coworkers (Lundberg et al., 2008) summarized the importance of the nitrate–nitrite–NO pathway emphasizing that it works partly parallel with the L‐arginine–eNOS pathway. The activity of eNOS is reduced in case of hypoxia, when the nitrite-induced increase in NO bioactivity becomes more important.

Thus it seems that in general, L-Arg does not improve CBF, but in certain selected conditions L-Arg can be used in patients to reduce systemic blood pressure and improve coronary circulation without major side effects. These conditions need to be clarified and investigated in future studies in order to include them in the guidelines.

### Functional Importance of Nitric Oxide and Nitric Oxide Synthase in the Regulation of Coronary Circulation

The regulation of coronary circulation and blood flow distribution in the coronary arteries are accomplished through the regulation of the tone and the resistance of coronary microcirculation. Coronary vascular resistance is the most important factor in determining CBF (Reitsma et al., 2007). Endothelium-derived vasoactive derivatives, metabolic factors, and neuro-hormones deliver the vasodilator and vasoconstrictor signals that should be balanced to maintain proper coronary vascular tone (Vanhoutte et al., 1986; Padro et al., 2020).

Since the identification of the endothelium-derived relaxing factor by Furchgott and Zawadzki (1980) hundreds of studies were conducted to elucidate the relationship between NO and CBF in healthy and diseased conditions. These studies revealed that myocardial perfusion is regulated by eNOS-synthetized NO in the coronary arteries. It is of note that these results are based on the effects of methylated L-arginines, because it is complicated to detect the level of NO *in vivo*. Inhibition of NO synthesis reduced CBF and enhanced the vulnerability of the myocardium to ischemia (Padro et al., 2020).

Many experiments were conducted on isolated large vessels, showing that L-Arg, ACh, and other agonists (bradykinin, substance P, etc.) elicit relaxations of smooth muscle *via* release of NO from the endothelium (Cherry et al., 1982). Less, if any, were known, whether or not the endothelium of small resistance vessels is also able to produce NO, thereby contributing to the regulation of vasomotor tone and peripheral vascular resistance. Further, *in vivo* studies showed that small arterioles (~20 µm in diameter) also dilate to ACh which is mediated by endothelium-derived NO (Koller et al., 1989). Later studies also demonstrated that isolated coronary arterioles (~150 micron) dilated in response to various endothelium dependent agonists and increases in wall shear stress by producing NO (Ungvari et al., 2002). Ungvari et al. (2002) demonstrated that NO is the main mediator in coronary circulation of rats, because L-NNA completely eliminated the dilation to increases in wall shear stress.

The first step of mechanotransduction is the activation of endothelial glycocalyx (GC) (Hein et al., 2003), which is a dynamic layer covering the luminal surface of endothelial cells (van den Berg et al., 2003). It comprises a network of glycoproteins, proteoglycans, and glycosaminoglycans (GAGs). Heparan sulfate, chondroitin sulfate, and hyaluronan are the major GAG constituents. Their volume and composition are actively regulated by the endothelial cells; and they proved to be tissue- and vessel-specific. The volume of GC in healthy individuals is estimated to be 1.5–1.7 L. It functions to protect vascular integrity by regulating vascular wall permeability and hemostasis and by possessing anti-atherogenic and anti-inflammatory properties. It is also essential for the flow-dependent NO production. In response to shear stress adaptive increase occurs in the amount of GC (Reitsma et al., 2007; Olde Engberink et al., 2015). The stretch of mechanotransducer glycocalyx-lipid bilayer-cytoskeleton system activates mechanosensitive ion channels, enhances Ca^++^ influx and NO production that results in vasorelaxation (Dragovich et al., 2016). A further mechanism for the GC-mediated NO generation is related to its negatively charged GAG components that serve as sodium buffer/barrier. When the amount of GC is markedly reduced, these protective functions are compromised and the sodium load reaches the cell surface, induces the expression and activity of endothelial sodium channels that allow sodium influx into the cells. Sodium loaded endothelial cells stiffen, their NO production impairs resulting in increased vascular tone (Kusche-Vihrog et al., 2014).

Degradation of GC and shedding of its components is commonly observed in certain clinical conditions including inflammation, atherosclerosis, ischemia, diabetes mellitus, chronic renal failure, and major vascular surgery (Mulivor and Lipowsky, 2004; Rehm et al., 2007). With these observations in line, Dekker et al. have noted that in patients who underwent on-pump coronary artery bypass graft surgery the early postoperative microcirculatory perfusion disturbances were associated with elevated plasma levels of heparan sulfate and syndecan-1, the markers of GC shedding. Moreover, they provided evidence for the prolonged postoperative impairment and for the delayed recovery of the functional integrity of the vascular walls after on-pump surgery (Dekker et al., 2019). Therapeutic attempts to preserve and restore GC with highly purified mixture of its constituents (sulodexide) are promising, however, further studies are warranted to draw definitive conclusions (Broekhuizen et al., 2010).

Various data from experimental and human studies completed during the last three decades demonstrated that the deterioration of NO-synthesis and bioavailability has a pivotal role in the development of several cardiovascular diseases. This is due to the fact that NO also inhibits thrombocyte aggregation and adhesion, resulting in the prevention of coronary circulatory dysfunction, thrombosis, and atherosclerosis (Toda and Toda, 2011). Reduced expression of NO synthase (Dornyei et al., 2015), increased production, and function of NOS inhibitors, NO scavengers, and vasoconstrictor substances are the most important mechanisms causing endothelial dysfunction. Decreased expression and function of NOS, reduced NO bioavailability, and enhanced generation of oxygen radicals may also play a pivotal role in the deterioration of endothelial function. Superoxide production is determined mainly by nicotinamide adenine dinucleotide phosphate (NADPH) oxidase, xanthine oxidase, and NOS uncoupling. Risk factors of cardiovascular diseases including smoking, obesity, aging, hyperglycemia, hypercholesterolemia and hypertension and excessive salt intake can also lead to impaired endothelial function (Hadi et al., 2005).

#### Effects of Methylated L-Arginines

Lefroy et al. provided the first evidence for a role of NO in the regulation of human coronary circulation (Lefroy et al., 1993). L-NMMA, an L-arginine analogue that specifically inhibits NOS, was used to inhibit the synthesis of NO in patients without CAD as proven by coronary angiography. Intracoronary infusion of L-NMMA decreased basal distal left anterior descending coronary artery diameter and basal CBF. L-NMMA did not alter heart rate and systemic blood pressure throughout the infusions, ruling out confounding hemodynamic factors. These observations clearly demonstrated that there is a small basal release of NO in the distal epicardial coronary arteries and resistance vessels (Lefroy et al., 1993). The role of NO in the control of vasomotor tone in human coronary arteries under resting conditions has been confirmed by several groups (Quyyumi et al., 1995a; Quyyumi et al., 1995b; Egashira et al., 1996a; Nishikawa and Ogawa, 1997; Quyyumi et al., 1997a; Tousoulis et al., 1997; Duffy et al., 1999; Seddon et al., 2009; Shabeeh et al., 2013).

Previously, substance P was shown to elicit endothelium-dependent relaxation *via* release of endogenous NO in isolated human epicardial coronary arteries (Chester et al., 1990). Local administration of substance P resulted in both epicardial and microvascular coronary vasodilatation in patients, and these effects were suppressed by L-NMMA (Quyyumi et al., 1997b). Notably, substance P-induced coronary dilation was attenuated in patients with hypertension and hypercholesterolemia, although there was no sign of coronary atherosclerosis during angiography. This may suggest the development of endothelial dysfunction under these conditions (Quyyumi et al., 1997b).

#### Mechanisms Which Reduce Endothelial Nitric Oxide Production

##### Insulin Sensitivity

Clinical and experimental studies have indicated that insulin plays an essential role in the regulation of endothelial function (DeFronzo and Ferrannini, 1991; Muniyappa et al., 2007). It operates in two opposite ways. On the one hand it is vasoprotective; stimulates endothelial NO generation by activating the phosphoinositide 3-kinase (PI3-K)-AKT pathway that induces the expression and activation of NOS. On the other hand, it activates the mitogen activated protein kinase (MAPK)–dependent signaling pathway that regulates the excretion of vasoconstrictor ET-1. Under physiological conditions these opposing endothelial effects of insulin are in balance. However, in pathologies associated with insulin resistance insulin signaling is directed toward the MAPK-ET-l pathway, whereas the PI3-K-NO pathway is markedly reduced. This imbalance may lead to endothelial dysfunction characteristic of insulin resistant states and may progress to the remodeling of vascular wall and atherosclerotic lesions (Muniyappa and Sowers, 2013; Janus et al., 2016).

Oxidative and inflammatory stress and the enhanced activity of renin-angiotensin-aldosterone system (RAAS) may contribute to the predominance of the MARK-ET-1 over PI3-K-Akt-NO pathway. It is particularly relevant to patients with coronary artery diseases who encounter tissue hypoxia- related high-levels of reactive oxygen species and pro-inflammatory molecules which are further augmented by their surge in response to ischemia-reperfusion at revascularization intervention (Gao et al., 2008). To improve/re-establish insulin sensitivity in patients with insulin-resistant endothelial dysfunction pharmacotherapy (thiazolidinediones, metformin, rosiglitazone) and lifestyle modifications are being implemented.

#### Ischemia/Reperfusion Injury: Inducible Nitric Oxide Synthase

**Figure 3:**
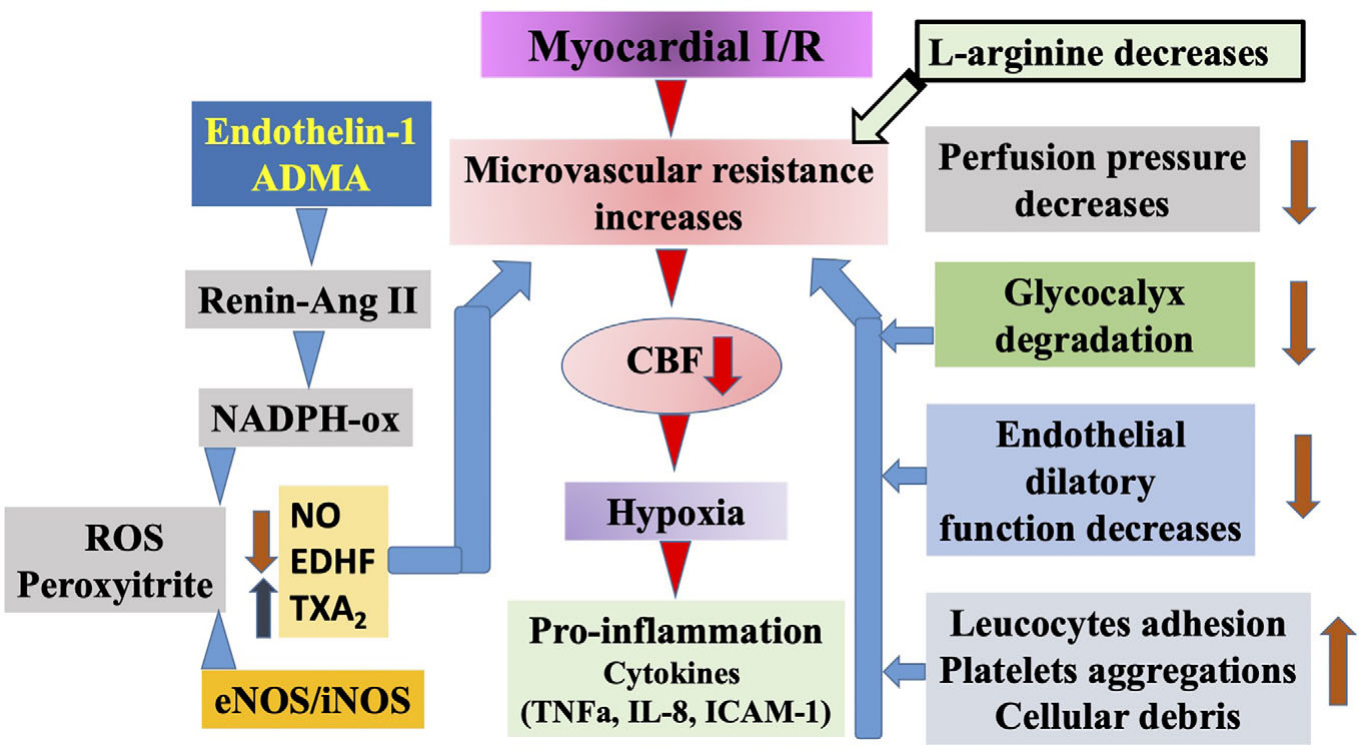
Schematic illustration of ischemia/reperfusion (I/R) injury of the myocardium. Prolonged ischemia reperfusion elicits serious injury to the microvascular segment of coronary circulation. Among others, perfusion pressure decreases, glycocalyx degrades, endothelium became impaired, and increased leucocyte adhesion as well as platelet aggregation occurs. Moreover, during ischemia some of the endothelial cells become necrotic and together with other cells produce debris. All of these events reduce the diameter of microvessels’ lumen. In addition, reperfusion initiates a series of events, including increased level of endothelin-1 and asymmetric dimethylarginine (ADMA), the latter activates the vascular renin angiotensin system followed by activation of nicotinamide adenine dinucleotide phosphate (NADPH)-oxidase and increased superoxide as well as peroxynitrite production, and increases constrictor prostanoid level, all of which constrict microvessels. In subsequent pro-inflammatory conditions cytokines are produced, initiating a full inflammatory response, apoptosis, and necrosis. Elevated levels of ADMA contribute to these events by blocking and uncoupling endothelial nitric oxide synthases (eNOS)/inducible NOS (iNOS) leading to superoxide production and thus exogenous L-arginine may provide beneficial effects.

Ku reported first that vasodilatation of coronary arteries is impaired after ischemia/reperfusion (I/R) injury (Ku, 1982). Control of CBF and microcirculatory perfusion is the most important determinant of outcome after acute coronary syndrome or I/R injury of the myocardium. Although the lack of NO can lead to decreased CBF and imperfect myocardial perfusion, the increased production of NO—because of the enhanced activation of inducible nitric oxide synthase (iNOS)—can result in excessive production of free-radicals thus myocardial damage. In an experimental model of circumflex coronary artery occlusion, inhibiting the activation of iNOS led to improved CBF and myocardial perfusion, while the increased function of the enzyme during 48 h of reperfusion resulted in an I/R injury in rabbits. Long-standing regional ischemia caused by LAD occlusion in a swine model increased the expression of iNOS in the myocardium. Enhanced iNOS activity may have a role in contractile dysfunction through the increased nitrite contents. The bioavailability of L-Arg is also very important in maintaining the normal vascular resistance *via* the regulation of NO bioavailability as it can decrease the serum level of soluble adhesion molecules and may also enhance the reaction of the endothelium after I/R (**Figure 3**).

#### Exercise, Nitric Oxide Synthase, and Nitric Oxide

Myocardial oxygen demand increases during physical exercise or cardiac pacing. Normally, CBF increases because of the vasodilatation of human coronary arteries (Laughlin et al., 2012). Growing evidence suggests that NO contributes to microvascular dilation and epicardial vasodilation in response to metabolic stimulation with atrial pacing in patients with normal or almost normal coronary arteries (Quyyumi et al., 1995a; Tousoulis et al., 1997; Duffy et al., 1999; Shabeeh et al., 2013). However, others reported that L-NMMA inhibited the pacing-induced dilatation of the large coronary arteries, but pacing-induced increases in CBF did not change (Egashira et al., 1996b; Nishikawa and Ogawa, 1997), suggesting that the involvement of NO in metabolic vasodilatation may differ between large epicardial and resistance coronary arteries. Importantly, coronary dilation in the face of increased metabolic demand is significantly attenuated in the presence of risk factors for coronary atherosclerosis or definitive CAD (Quyyumi et al., 1995a; Nishikawa and Ogawa, 1997; Tousoulis et al., 1997).

The complexity of the effect of NO is highlighted by Berstein et al., who in conscious dogs determined the NO production during exercise. They used nitro-L-Arg (NLA) to block NOS and measured several hemodynamic parameters during exercise. As an index for NO they measured plasma nitrate/nitrite, glucose, lactate, and free fatty acid (FFA) levels. Exercise increased NO production, which was blocked by L-NNA. Also, in the presence of L-NNA myocardial oxygen consumption increased significantly, reduced FFA consumption, indicating that NO influences myocardial substrate metabolisms (Bernstein et al., 1996).

#### Perivascular Nerves and Neuronal Nitric Oxide Synthase

eNOS has been considered as the major source of endothelium-derived NO causing local increases in blood flow (Vanhoutte et al., 2016). However, perivascular nerves and cells within the vessel wall express neuronal NOS (nNOS) (Toda and Okamura, 2003; Webb et al., 2006), and local nNOS-derived NO may influence vascular tone. Indeed, an nNOS-selective inhibitor, S-methyl-L-thiocitrulline (SMTC), reduced basal CBF, and epicardial coronary diameter (Seddon et al., 2009). Recently, it has been established that nNOS plays a role in mental stress-induced vasodilatation in human coronary circulation (Khan et al., 2017). In contrast, SMTC had no effect on substance P- and pacing-induced increases in CBF, although these responses were significantly attenuated by the nonselective NOS inhibitor L-NMMA, suggesting the involvement of eNOS but not nNOS (Seddon et al., 2009; Shabeeh et al., 2013).

These data show that eNOS and nNOS play different local roles in the control of human coronary circulation *in vivo*. However, further studies are warranted to explore how the relative contribution of eNOS and nNOS may be modified in certain diseases.

#### Nitric Oxide in Human Therapy: Lost in Translation?

Despite great success in basic science regarding the mechanism and function of NO in the control of coronary circulation and maintaining the healthy condition of large vessels, there is little if any advancement in clinical areas. Already 400 years earlier, Tao Hongjing *(* http://www.goldenelixir.com/taoism/tao_hongjing.html *)* used KNO_3_, saltpeter to reduce the angina and treat CVD of patients. In the last century the most popular antianginal agents were nitroglycerin, sodium trioxodinitrate (Angeli’s salt), isosorbide mononitrate, isosorbide dinitrate, nitroglycerin transdermal patch. The clinical use of organic nitrite and nitrate esters is limited due to the fact that increased angiotensin II-dependent generation of superoxide anions can be observed from NAD(P)H oxidase and eNOS (Ignarro et al., 2002). They have also been shown to have mutagenic effects.

Organic nitrates are a heterogeneous group of vasodilators (Gori and Daiber, 2009). There are some progresses in the development of organic nitrates to find new therapeutic applications. In the study of Lehmann and Daiber, the mononitrate aminoethyl nitrate (AEN) present an almost similar potency compared to glyceryl trinitrate (GTN) (Schuhmacher et al., 2009). In contrast to triethanolamine trinitrate (TEAN) and GTN, the activation of AEN was not dependent on mitochondrial aldehyde dehydrogenase (ALDH-2) and AEN did not cause *in vitro* tolerance. *In vivo* treatment with TEAN and GTN, but not with AEN led to cross-tolerance to ACh-dependent or to GTN-dependent vasodilatation. All nitrates examined induced tolerance, but only TEAN and GTN significantly enhanced mitochondrial oxidative stress *in vitro* and *in vivo*. This study demonstrated that high potency nitrates do not automatically induce oxidative stress or tolerance. It has been also documented that not all the high potency nitrates are activated by ALDH-2 (Daiber et al., 2010).

In pulmonary hypertension, NO as a gas is used to reduce mean pulmonary arterial pressure, and pulmonary vascular resistance index, indicating reduced vascular resistance (Atz et al., 1999), however, in coronary circulation it did not become a part of regular treatment modalities. At present, inhaling or administration of NO in other ways is not used routinely to improve coronary circulation in humans. Thus, future innovation may help to find a way to use this multipurpose molecule to improve the function of coronary circulation and the heart.

### Overview of Biosynthesis, Metabolism, Excretion, and Actions of Asymmetric Dimethylarginine

#### Generation of Asymmetric Dimethylarginine Methylation of L-Arginine: Asymmetric Dimethylarginine, Symmetric Dimethylarginine

ADMA is synthesized by protein arginine methyltransferase type I (PRMT-1) (Boger, 2005). It has been demonstrated that ADMA inhibits the catalytic function of NOS thus decreases the release of NO and suppress the vascular effects of NO (Ogawa et al., 1989; Moncada and Higgs, 1993; Ueda et al., 2003). L-Arg is a substrate for both NOS and the PRMT-1 thus inducing ADMA generation through methylation of L-Arg (Vallance and Leiper, 2004). ADMA is primarily metabolized by the enzyme DDAH to citrulline and dimethylamine, and the main route of elimination is *via* renal excretion (Boger, 2004; Pope et al., 2009).

#### Elimination of Asymmetric Dimethylarginine: Dimethylarginine Dimethylaminohydrolase

DDAH-1 has been identified to be the main isoform with nNOS function in tissues, while tissue presence of DDAH-2 primarily overlaps with the expression of eNOS and iNOS (Palm et al., 2007; Tousoulis et al., 2015). For this reason, metabolism of ADMA in the brain, where only nNOS can be observed, is mainly done by DDAH-2. On the other hand, DDAH-2 plays a pivotal role in the degradation of ADMA in cardiovascular and immune tissues, where eNOS and iNOS are present in a greater extent (Palm et al., 2007). ADMA is mainly metabolized in the kidney and the liver (Nijveldt et al., 2003a; Nijveldt et al., 2003b).

### Asymmetric Dimethylarginine and Regulation of Microvascular Resistance

To provide biological proof for the idea that endogenous ADMA indeed may have substantial effects on blood flow we have administered ADMA into isolated microvessels (160 µm at 80 mmHg intraluminal pressure), a condition in which all other confounding factors have been excluded. We have found that ADMA elicited a dose dependent decrease in basal diameter (myogenic tone), and inhibited wall shear stress-induced NO release and dilation (Toth et al., 2007). In addition, ADMA induced inhibitor responses could be eliminated by SOD, apocynin ACE inhibitor quinapril, or the angiotensin II receptor type 1 (AT1) receptor blocker losartan indicting that ADMA activates angiotensin II-NAD(P)H oxidase pathway, which is then responsible for an increased superoxide production (indicated by ethidium bromide fluorescence), interfering with NO released from NO donor or increases in wall shear stress, but it did not affect the dilator responses to the calcium channel blocker nifedipine or 8-bromo cGMP indicating its selective action on NOS (Veresh et al., 2008; Veresh et al., 2012). On the basis of these findings it can be proposed that endogenous MLAs are regulators of NO-mediated dilations both by inhibiting NOS and *via* superoxide production interfering with NO.

Other authors also found that ADMA—in addition, to be a competitive inhibitor of eNOS—activates the RAS-Ang II pathway, which then generates reactive oxygen species (ROS). ROS by interacting with NO reduces the bioavailability of NO leading to reduced SMC relaxation/constriction thus impaired coronary circulation (Toth et al., 2007; Veresh et al., 2008; Veresh et al., 2012).

### Asymmetric Dimethylarginine, Cardiac Remodeling, and Coronary Revascularization

Potential Pathological Roles of Endogenous Methylated L-Arginines.

The relationship between increased ADMA level and mortality or major adverse cardiovascular events has been documented by several prospective clinical studies (Moncada and Higgs, 1993). For that reason, ADMA may be useful as a diagnostic tool and as a risk marker (Vallance et al., 1992). ADMA plays an important role in the control of coronary vascular function. Cable et al. (2009) observed that endothelium-dependent vasodilatation is less pronounced in the coronary arteries than in the femoral and renal arteries.

### Asymmetric Dimethylarginine and Cardiovascular Diseases—Meta-Analyses

Large clinical trials have emphasized the prognostic significance of ADMA, because there is a considerable relationship between elevated ADMA concentration and adverse cardiovascular events. A meta-analysis of Willeit et al. involving 19,842 patients registered 2,339 CVD, 997 cases coronary heart disease, and 467 stroke during a mean follow-up of 7.1 years (Willeit et al., 2015). They reported a significant association between elevated baseline serum ADMA concentration and all the outcomes mentioned before. The combined risk ratios were 1.42, 1.39, and 1.6 for CVD, coronary heart disease, and stroke, respectively (95% confidence intervals were 1.29 to 1.56, 1.19 to 1.62, and 1.33 to 1.91 for CVD, coronary heart disease, and stroke, respectively). Moreover, ADMA also seems to be an important predictor of cardiovascular death in patients with high-, intermediate-, and low cardiovascular risk (Boger et al., 2009). The relationship between increased ADMA serum concentration and congestive heart failure has been proposed by experimental and human studies raising the suspicion that ADMA might have an etiological role in the pathogenesis of heart failure (Feng et al., 1998).

Seljeflot et al. pointed out that the L-Arg/ADMA ratio influenced the severity of heart failure more than serum ADMA concentration did (Seljeflot et al., 2011); this may indicate that the competitive inhibition of eNOS is the most important mechanism leading to this association (Tousoulis et al., 2015). Intravenous administration of low dose ADMA has been reported to decrease heart rate and cardiac output and increase blood pressure (Achan et al., 2003). However, enhanced oxidative stress present in congestive heart failure can reduce DDAH and increase PRMT 1 activity thus resulting in elevated ADMA serum concentration (Searles, 2002).

A meta-analysis including 16 case–control studies of 4,713 participants investigated the relationship between the serum ADMA concentration and the risk of CAD (Xuan et al., 2016); 2,939 patients and 1,774 control subjects were examined in the meta-analysis. ADMA concentration was significantly higher in patients than in control subjects (Weighted Mean Differences: 0.248, 95% CI: 0.156–0.340; p = 1.16 e–7), suggesting that ADMA might be a risk factor for CAD. The subgroup analysis proved that ADMA serum level was similarly elevated in the different types of CAD, i.e., in patients with myocardial infarction [weighted mean difference (WMD): 0.397, 95% CI: 0.112–0.683; p = 0.0106], stable angina pectoris (WMD: 0.197, 95% CI: 0.031–0.364; p = 0.02), and unstable angina pectoris (WMD:0.857, 95% CI: 0.293–1.420; p = 0.003).

Willeit and coworkers observed that in the meta-analysis of 22 prospective studies, there were 2,339 cases of CVD, 997 coronary heart disease, and 467 stroke during the mean follow-up of 7.1 years (Willeit et al., 2015). When compared to subjects in the bottom third of baseline ADMA level, those in the top third of baseline ADMA concentration were identified at a 40% elevated risk of CVD. This association was similar in participants with and without preexisting CVD or kidney disease at baseline and across studies that used diverse methods to measure dimethylarginine levels. On the basis of somewhat limited data available on symmetric dimethylarginine (SDMA), no significant association between SDMA concentration and the risk of cardiovascular outcomes could be observed.

#### Asymmetric Dimethylarginine Levels and Atherosclerosis

Mangiacapra and coworkers demonstrated that serum ADMA concentration significantly correlated with the presence and extent of coronary atherosclerosis in subjects undergoing elective coronary angiography (Mangiacapra et al., 2016). Measurement of fractional flow reserve (FFR) was performed in case of at least one intermediate coronary artery stenosis (≥50% diameter stenosis). Blood samples were harvested for the detection of ADMA serum concentration before coronary angiography. They found that ADMA levels across tertiles were significantly correlated with both the stenosis score (2.25 ± 1.70 *vs*. 2.89 ± 1.99 *vs*. 2.95 ± 1.82, p=0.016) and the extent index (0.52 ± 0.32 *vs*. 0.61 ± 0.39 *vs*. 0.72 ± 0.47, p=0.003). The relationship between ADMA levels and extent index remained significant after multivariate adjustment (p = 0.005). FFR ≤0.80 represents the functional significance of coronary stenosis in CAD. The 113 patients having FFR ≤0.80 in at least one coronary artery had significantly increased ADMA concentrations compared to the subjects without functionally severe CAD [0.51 (0.43–0.64) *vs*. 0.46 (0.39–0.58) μmol/l, p = 0.005]. Serum ADMA concentration was independent predictors of abnormal FFR after adjustment for extent score (odds ratio 7.35, 95% confidence interval 1.05–56.76, p = 0.046). Furthermore, patients with the largest atherosclerotic burden and FFR ≤0.80 had the highest serum ADMA concentration. These data also emphasize the relationship between increased ADMA concentration and the presence and extent of coronary stenosis as diagnosed with abnormal FFR. This association might explain the fact that patients with elevated serum ADMA concentration carry high cardiovascular risk.

### Asymmetric Dimethylarginine and Coronary Circulation

#### Asymmetric Dimethylarginine Levels and Coronary Artery Disease

In response to myocardial ischemia, coronary angiogenesis and collateral vascular growth start to restore CBF and save myocytes in the ischemic myocardium. The new collateral arteries are especially important in patients with significant stenosis of a large coronary artery (Habib et al., 1991).

NO has been proved to play a pivotal role in angiogenesis. The function of the eNOS was an important determinant of angiogenesis in experimental studies (Murohara et al., 1998). NO also increases the activity of the vascular endothelial growth factor, which is confirmed to be one of the main mediators in collateral growth.

Decreased NO production caused by elevated serum concentration of ADMA may not be the only mechanism leading to inadequate coronary collateral growth. Elevated plasma level of ADMA may enhance the activity of ACE thus the function of RAAS (Suda et al., 2004). The abnormal activation of RAAS may increase the production of vasoconstrictor agents. These vasoconstrictor factors may reduce the formation of collateral vessels and further decrease NO generation. Kocaman and coworkers investigated the relationship between serum ADMA concentration and the growth of coronary collateral system (Kocaman et al., 2008). They proved that patients with proper collateral formation had lower plasma ADMA concentration compared to those subjects who did not develop good coronary collateral vessels (0.41 ± 0.25 µmol/l *vs*. 0.70 ± 0.23 µmol/l, p =0.001). The serum ADMA concentration of patients with good collateral system was similar to those who had normal coronary arteries.

They also found that higher L-Arg/ADMA ratio was related with better coronary collateral formation. In conclusion, their findings suggest that higher serum ADMA concentration is associated with poor coronary collateral growth. ADMA might have a key role in coronary collateral development and might explain the differences among different subjects with CAD. Inhibitors of NO might be important in all steps of coronary collateral formation as they are determinative factors in the function of the endothelial cells.

Cardiac allograft vasculopathy is a leading cause of chronic graft deficiency after heart transplantation (Parikh et al., 2017). ADMA has been suggested to play a role in maintaining proper coronary circulation following transplantation and also in the progression of cardiac allograft vasculopathy.

#### Asymmetric Dimethylarginine Levels Correlate With the Severity and Extent of Coronary Artery Disease in Patients With Stable Coronary Artery Disease

Interestingly, previous studies investigating the relationship between ADMA concentration and coronary atherosclerosis have documented divergent results (Mangiacapra et al., 2016). ADMA concentration was higher in patients with significant coronary stenosis (≥50% in one major coronary artery) on coronary angiography when examining subjects with stable CAD (Lu et al., 2003a). There was also a significant correlation between ADMA concentration and the severity and the extent of atherosclerotic coronary artery stenosis (number of vessels with ≥50% stenosis) (Thum et al., 2005; Schulze et al., 2006). In the study of Kruszelnicka et al. (2013), a significant positive correlation between ADMA concentration and the extent of CAD as evaluated with the Sullivan score was observed. These observations were only partly confirmed by later studies. Discrepancies might be the result of different threshold values (e.g., ≥20%) used to define the severity of coronary artery stenosis on coronary angiography and the application of distinct score systems (e.g., Friesinger score) (Meinitzer et al., 2007) or by including patients with acute coronary syndromes (Borgeraas et al., 2012). Indeed, it has been confirmed that ADMA serum concentration is higher in patients with acute coronary syndrome (Boger et al., 2009). For this reason, including patients with acute myocardial infarction may lead to conflicting results in a study trying to find a relationship between ADMA concentration and the extent of CAD.

#### Correlation Between Asymmetric Dimethylarginine Levels and Coronary Fractional Flow Reserve

In the study of Parikh et al., FFR, the index of microcirculatory resistance and coronary flow reserve were measured with a pressure wire in the LAD at baseline (within 8 weeks) and 1 year after heart transplant in 46 patients (Parikh et al., 2017). There was a significant inverse relationship between ADMA concentration and FFR values at baseline (r = −0.33; p = 0.024), which persisted on year after transplant (r = −0.39; p = 0.0085).

At baseline, patients with an FFR <0.90 (a prognostically validated cutoff) had significantly higher ADMA concentration (0.63 *vs*. 0.54 µM; p = 0.034). After multivariable adjustment, ADMA concentration (odds ratio 1.80 per 0.1 µM; 95% confidence interval 1.07 to 3.03; p = 0.027) was an independent predictor of FFR <0.90 at baseline. There was no significant relationship between ADMA level and the microcirculatory resistance index or coronary flow reserve. The authors concluded that high baseline ADMA concentration was an independent predictor of FFR <0.90, indicating a strong relationship between elevated serum ADMA level and epicardial dysfunction early after heart transplantation. Consequently, measurement of baseline concentration of ADMA may help to recognize patients with a higher risk of cardiac allograft vasculopathy. Furthermore, it has been recently documented that impaired FFR measured early after heart transplantation might be a predictor of late mortality or re-transplantation (Yang et al., 2016).

#### Time Dependent Changes in Plasma Asymmetric Dimethylarginine Level in Patients After Stent Implantation

Suzuki et al. reported that in patients with native CAD intramyocardial injection of L-Arg reduced the formation of neointimal hyperplasia after stent implantation (Suzuki et al., 2002). In addition, high level of ADMA in the plasma seems to be an independent predictor of later cardiovascular events after percutaneous coronary intervention (PCI) (Lu et al., 2003b). To clarify this issue, we conducted a clinical study to investigate the plasma concentration of ADMA in 30 patients who underwent PCI with stent implantation. Moreover, we studied the relationship among ADMA and L-Arg, SDMA, and L-ornithine levels (Ajtay et al., 2009).

Evidence has been found that restoration of coronary circulation by stent implantation elicited a rapid and maintained reduction in the level of ADMA and this reduced level was present during the 30-days follow-up period after stenting regardless what stent type were used. There was a significant time lapse by group interaction for ADMA (F=12.8, p<0.0001), SDMA (F=5.5, p=0.013). Furthermore, L-ornithine showed a significant decrease after stent placement (F=12.5, p<0.0001). In contrast, the plasma level of L-Arg increased significantly. All together these changes increased the L-Arg/ADMA ratio which then did not change. The physiological importance of the increased ratio is that it may increase the activity of eNOS, which by producing more NO can improve coronary circulation (Suzuki et al., 2002; Bode-Boger et al., 2007) by promoting the revascularization process (Luque Contreras et al., 2006). In clinical settings it means that the general condition of myocardium improves and several indices, such cardiac output, ejection fraction, and myocardial contractility improves, as well as the short- and long-term morbidity and mortality (Investigators, G. A., 1993; Stone et al., 1998).

#### Post-Stenting Changes in the Plasma Concentrations of L-Arginine, Asymmetric Dimethylarginine, and Symmetric Dimethylarginine as a Function of Time in Patients With Segment Elevation Myocardial Infarction

**Figure 4:**
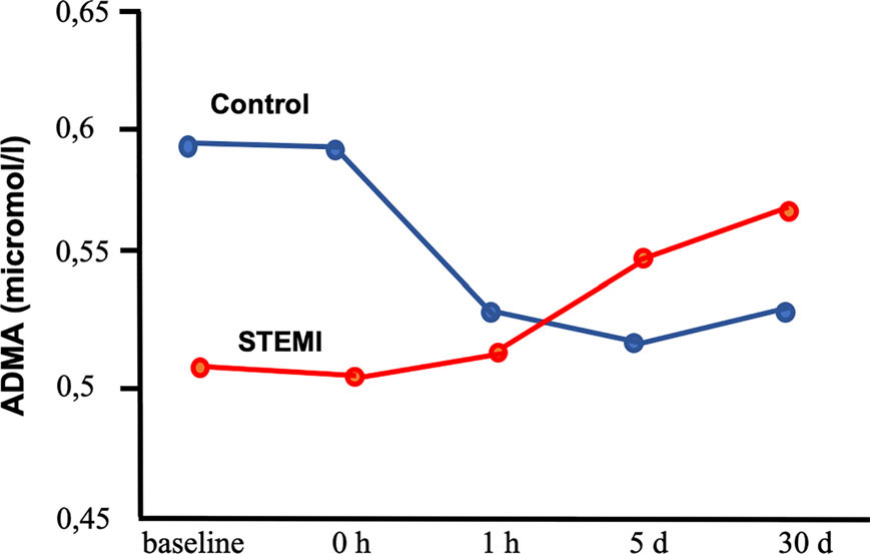
Plasma levels of asymmetric dimethylarginine (ADMA) in patients with coronary artery disease undergoing percutaneous coronary intervention with stent placement with or without (control group) ST segment elevation myocardial infarction (Ajtay et al., 2010). Segment elevation myocardial infarction (STEMI) group indicates patients with ST segment elevation myocardial infarction. Baseline: before stent placement. 0 h, 1 h, 5 days, 30 days: 0 h, 1 h, 5 days and 30 days after stent placement.

The standard therapy for acute ST segment elevation—indicating myocardial infarction—is PCI. Thus in order to see the importance of L-arginine and its derivatives we measured the plasma level of L-Arg, ADMA, and SDMA (**Figure 4**) immediately after PCI (Ajtay et al., 2010).

Interestingly, we have found a great difference in change of L-Arg and its methylated forms and L-ornithine among patient who had segment elevation myocardial infarction (STEMI) or not. Without STEMI there were significant reductions of ADMA and L-ornithine, whereas L-Arg increased. These changes increased the ratio of L-Arg/ADMA. In contrast, patient with STEMI showed a variety increase in L-Arg, methylated-L-Arg, and L-ornithine levels. In STEMI patients stent implementation elicited increases in L-Arg, ADMA, SDMA, and L-ornithine, but Arg-MI level became reduced at early time period and then increased back to its initial level. Evaluated by intersubject time effect, the changes in ADMA, MMA, Arg-MI, and L-ornithine proved to be significant between STEMI and non-STEMI groups. The findings of our study suggest that continuous impairment of endothelial function is not reduced by stenting STEMI patients. Although the reasons for such time dependent changes are not exactly known, one can hypothesize several underlying mechanisms. In STEMI patients the reduction in the level of ADMA did not occur suggests that in the infarcted area, still oxidative stress and proinflammatory processes are taking place initiated by ischemia/reperfusion. In contrast, plasma L-Arg level were similar in STEMI and non-STEMI patients. We suggest that this is due to reduced cellular uptake of L-Arg (Bae et al., 2005) and/or increased proteolysis (Chia et al., 2009) resulting in release of free amino acids, such as L-Arg and methylated-arginines.

#### Coronary Revascularization With or Without Cardiopulmonary Bypass Changes Differently Plasma Asymmetric Dimethylarginine Levels

**Figure 5:**
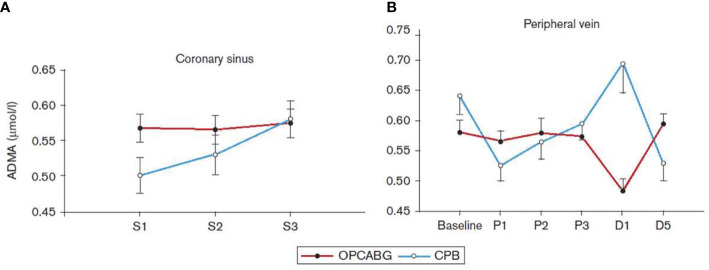
Serum concentration of asymmetric dimethylarginine (ADMA) collected from the coronary sinus **(A)** and from the peripheral vein **(B)** in patients with coronary artery disease undergoing coronary revascularization with cardiopulmonary bypass (CPB) or off-pump coronary artery bypass grafting (OPCABG). D1, the first postoperative day; D5, the fifth postoperative day; S1 and P1, after the insertion of a coronary sinus catheter; S2 and P2, after the completion of the first distal anastomosis; S3 and P3, after the completion of the last distal anastomosis (Cziraki et al., 2011).

Akila et al. reported that coronary artery bypass grafting (CABG) can be executed with extracorporeal perfusion with cardiopulmonary bypass, which results in global ischemia in the affected area of the heart. De-clamping leads to reperfusion injury, which is not only locally but in the whole body elicits inflammatory reactions (Akila et al., 2007). We extended our interest to patients who underwent CABG surgery with or without CPB and measured and compared serum levels of ADMA, SDMA, and L-Arg from coronary sinus (CS) and from peripheral veins (Cziraki et al., 2011). We collected intraoperative samples in which ADMA increased in the CPB patients for off-pump off pump CABG (OPCABG) and CPB patients (**Figure 5**). The peripheral blood samples also showed elevated ADMA level during CPB, whereas ADMA level did not changed during OPCABG.

The values of the L-Arg/ADMA ratio were significantly higher in the OPCABG group at baseline and on the first postoperative day compared with the results of the CPB group (178.29± 11.56 *vs*. 136.28 ± 13.72 and 129.43 ± 7.08 *vs*. 106.8 ± 6.9 for OPCABG and CPB groups, respectively).

All together the findings of these studies showed that plasma levels of ADMA, SDMA, L-Arg, and L-Arg/ADMA ratio indicate the presence of I/R injury, thus they can be viewed as markers of I/R injury in CABG patients. CPB caused severe I/R injury as shown by the increased ADMA level during operation and in the subsequent first operative day. As opposed to this, in the OPCABG patients the L-Arg/ADMA ratio decreased only in the first post-operation day. The underlying pathomechanisms could be that the increased level of oxidative stress activates PRMT-1 and at the same time inhibits DDAH, resulting in an elevated ADMA level. The high circulating ADMA level not only can inhibit eNOS activity but also uncouple this enzyme leading to further production of reactive oxygen metabolites (Lu et al., 2003a; Scalera et al., 2006).

#### Asymmetric Dimethylarginine and Endothelin in the Pericardial Fluid

**Figure 6:**
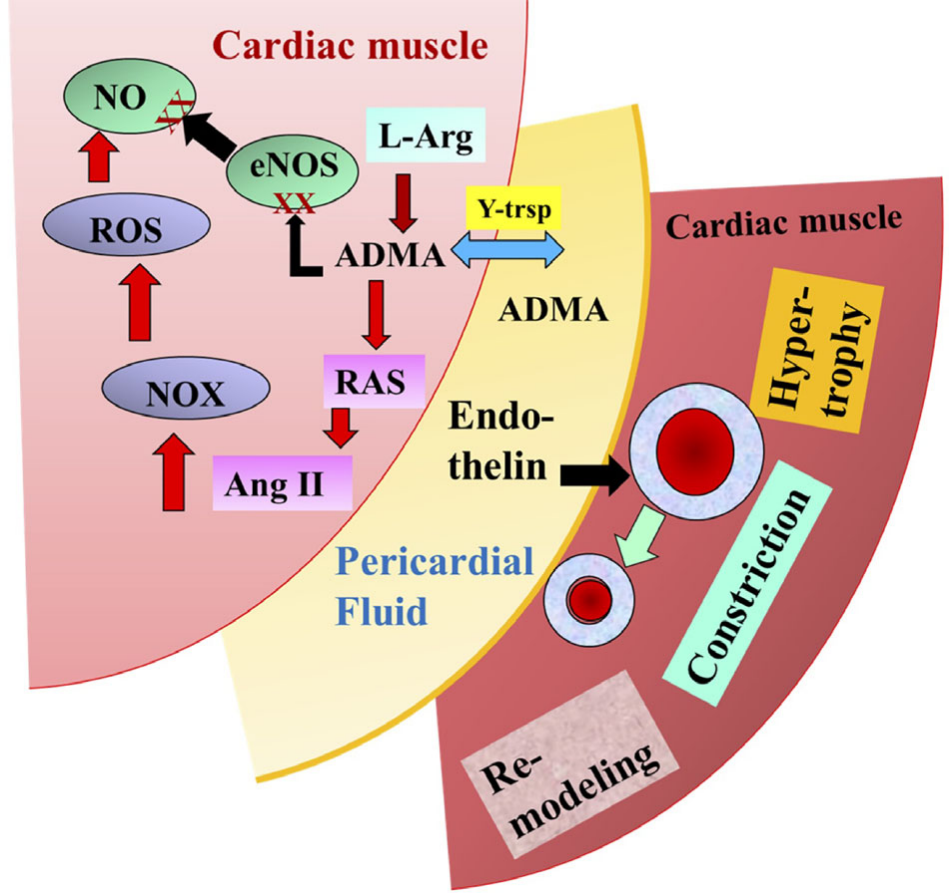
Illustrates the potential role of pericardial asymmetric dimethylarginine (ADMA) and endothelin in the modulation of coronary circulation and cardiac remodeling modified after Nemeth et al. (2015a).

For many years it was thought that the pericardial fluid (PF) has one primary role, namely to reduce the friction between the pericardium and the contracting heart. Recently, however, it was discovered that many biologically active molecules are also present in the PF (**Figure 6**) (Szokodi et al., 1998; Kawamoto et al., 2013). Nemeth et al. (2015a) investigated 28 patients undergoing CABG surgery, 25 undergoing valve replacement (VR) (Bosmans et al., 1999), and 20 non-cardiac patients (NCP). Plasma was collected from NCP, while both plasma and pericardial fluid were harvested from patients after median sternotomy. Since ADMA became elevated during cardiac surgery, it was hypothesized that ADMA is present in PF. Among others, in CABG patients but more so in valve replacement patients, the level of ADMA was high and correlated with hypertrophy of the left ventricle and other parts of the heart. There was a positive correlation between plasma L-Arg and ADMA in CABG (r = 0.539, p = 0.015), and plasma as well as PF L-Arg in CABG (r = 0.357, p = 0.031), plasma and PF ADMA in VR (r = 0.529, p = 0.003), PF L-Arg and ADMA in both CABG and VR (CABG: r = 0.05) (Nemeth et al., 2015a). There was also a negative correlation between ejection fraction and PF ADMA. Since ADMA is an inhibitor of NOS and an activator of RAAS it was concluded that ADMA contributes to the functional and morphological remodeling of the heart (Nemeth et al., 2015a). As discussed above and shown by the presence of reduced NO, the endothelin system became upregulated (Alonso and Radomski, 2003).

Indeed, our findings showed that the PF of CABG patient resulted in significant constriction of isolated arteries, which was inhibited by the selective inhibitor of endothelin A receptor, BQ123 (Nemeth et al., 2015b). Thus, PF being in direct contact with the larger coronary arteries on the surface of the heart can modulate their diameter and thus CBF. One can propose then, that during heart surgery PF and pericardial space can be used for therapeutic purposes.

## Conclusion

Since the discovery of the endothelium-derived relaxing factor by Furchgott and Zawadzki (1980) many investigations have been conducted both in basic science laboratories and clinical settings regarding the interaction between nitric oxide and coronary arteries or blood flow in healthy people and in patients with coronary artery disease. The present review covers recent developments in these investigations, including those reported over decades, on the roles of L-arginine and asymmetric dimethylarginine and endothelial and neural NOS in the control of coronary circulation in healthy humans and those with coronary artery disease or cardiac ischemia and reperfusion injuries. The discovery of the NO pathway resulted in a better understanding of the regulation of cardiovascular function and morphology and led to new possibilities for developing new pharmacological tools to improve the function of coronary circulation and the heart. Extensive clinical data support the notion that reducing asymmetric dimethylarginine concentration may provide a novel therapeutic approach in the treatment of some cardiovascular diseases, decreased nitric oxide synthesis being involved in disorders such as hypertension, atherosclerosis, and ischemia/reperfusion (Rochette et al., 2013). Moreover, convincing results confirm that serum asymmetric dimethylarginine concentration significantly correlated with the presence and extent of coronary atherosclerosis (Mangiacapra et al., 2016). Several exciting results have been published related to the early molecular part of these mechanisms, but there are also some conflicting findings, especially in the clinical translation. Similarly, great progress has been made in clarifying the mechanisms of coronary blood flow control in myocardial ischemia/reperfusion injury, however, we can see an apparent deficiency in the translation of these scientific data to clinical practice. This, however, should not deter young and old investigators, keeping in mind that a Nobel prize was given to topics related to nitric oxide pathway—acetylcholine and nitric oxide-donor—that have been “over-investigated” in previous decades. It is important to find the right conditions and the right set of patients where this vast knowledge can be used.

**Figure 7:**
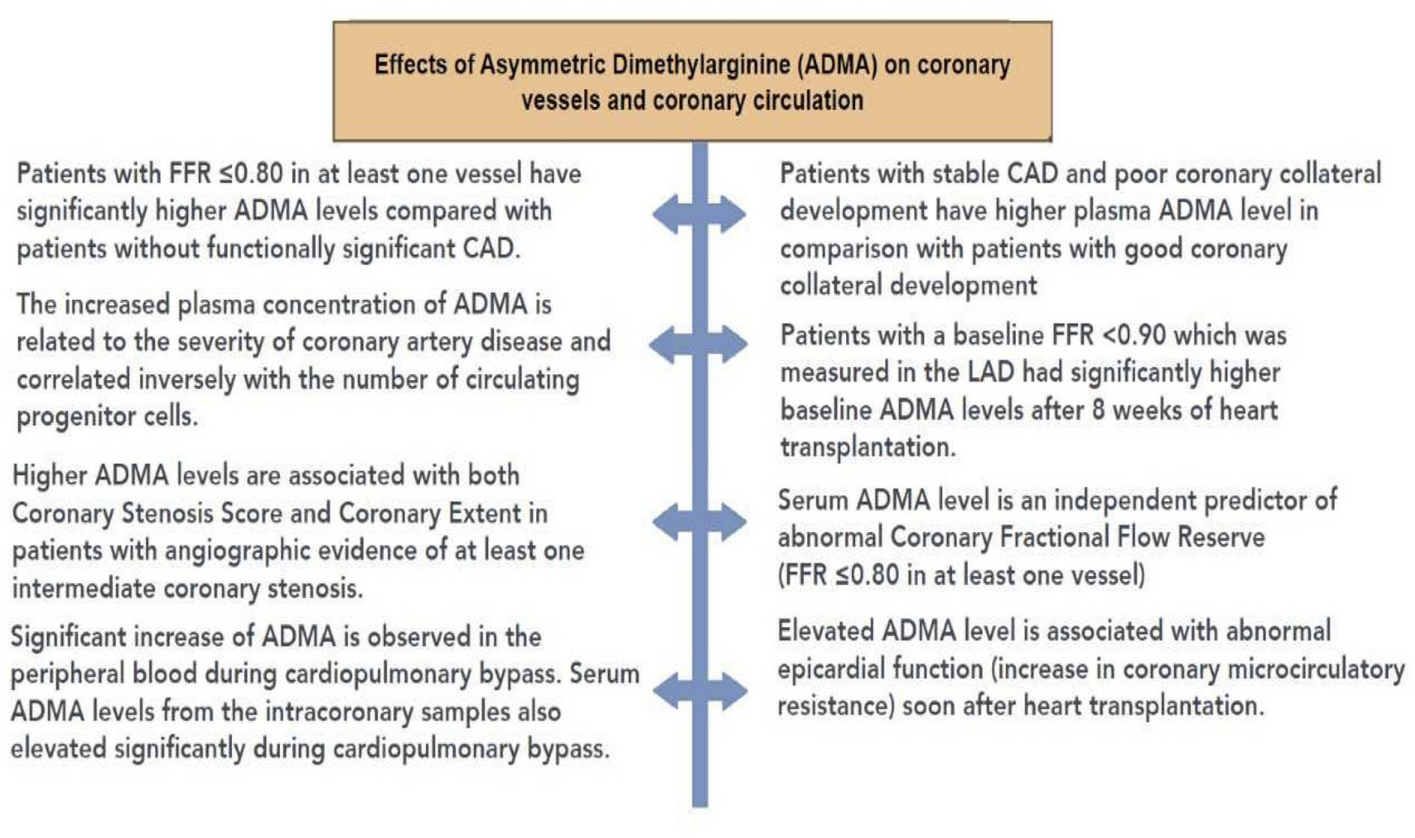
Summary of the effects of asymmetric dimethylarginine (ADMA) in human studies. ADMA, asymmetric dimethylarginine; CAD, coronary artery disease; FFR, fractional flow reserve.

**Figure 7** summarizes the effects of ADMA observed in human studies.

Many aspects have not been discussed in this review such as sex differences (Vaccarino et al., 2013), perinatal origin of cardiovascular diseases (Barker et al., 2006; Vida et al., 2009), age related changes (Kiss et al., 2020; Ungvari et al., 2020), specific metabolic diseases (Bagi et al., 2006; Szerafin et al., 2006), its association with preeclampsia (Yuan et al., 2017), and compensatory mechanisms (Sun et al., 1992; Huang et al., 2002). It is very likely that these areas will provide new discoveries in the cardiovascular field and will contribute to a better treatment of patients.

## Author Contributions

All authors listed have made substantial, direct, and intellectual contribution to the manuscript and approved it for publication.

## Funding

Support: National Research, Development and Innovation Fund, OTKA K 132596, and Scientific Excellence Program 2019, at the University of Physical Education, Innovation and Technology Ministry, Hungary TUDFO/51757/2019-ITM, and the Higher Education Institutional Excellence Programme of the Ministry of Human Capacities, Hungary (20765-3/2018/FEKUTSTRAT FIKPII).

## Conflict of Interest

The authors declare that the research was conducted in the absence of any commercial or financial relationships that could be construed as a potential conflict of interest.

## References

[B1] Achan V. Broadhead M. Malaki M. Whitley G. Leiper J. MacAllister R. (2003). Asymmetric dimethylarginine causes hypertension and cardiac dysfunction in humans and is actively metabolized by dimethylarginine dimethylaminohydrolase. Arterioscler. Thromb. Vasc. Biol. 23 (8), 1455–1459. 10.1161/01.ATV.0000081742.92006.59 12805079

[B2] Agullo L. Garcia-Dorado D. Inserte J. Paniagua A. Pyrhonen P. Llevadot J. (1999). L-arginine limits myocardial cell death secondary to hypoxia-reoxygenation by a cGMP-dependent mechanism. Am. J. Physiol. 276 (5), H1574–H1580. 10.1152/ajpheart.1999.276.5.H1574 10330241

[B3] Ajtay Z. Scalera F. Cziraki A. Horvath I. Papp L. Sulyok E. (2009). Stent placement in patients with coronary heart disease decreases plasma levels of the endogenous nitric oxide synthase inhibitor ADMA. Int. J. Mol. Med. 23 (5), 651–657. 10.3892/ijmm_00000176 19360324

[B4] Ajtay Z. Nemeth A. Sulyok E. Cziraki A. Szabados S. Martens-Lobenhoffer J. (2010). Effects of stent implementation on plasma levels of asymmetric dimethylarginine in patients with or without ST-segment elevation acute myocardial infarction. Int. J. Mol. Med. 25 (4), 617–624. 10.3892/ijmm_00000384 20198311

[B5] Akila B. D’Souza Vishwanath P. D’Souza X. X. X. V. (2007). Oxidative injury and antioxidants in coronary artery bypass graft surgery: off-pump CABG significantly reduces oxidative stress. Clin. Chim. Acta 375 (1-2), 147–152. 10.1016/j.cca.2006.07.001 16904092

[B6] Alonso D. Radomski M. W. (2003). The nitric oxide-endothelin-1 connection. Heart Fail Rev. 8 (1), 107–115. 10.1023/a:1022155206928 12652164

[B7] Atz A. M. Adatia I. Lock J. E. Wessel D. L. (1999). Combined effects of nitric oxide and oxygen during acute pulmonary vasodilator testing. J. Am. Coll. Cardiol. 33 (3), 813–819. 10.1016/s0735-1097(98)00668-8 10080486

[B8] Bachetti T. Comini L. Francolini G. Bastianon D. Valetti B. Cadei M. (2004). Arginase pathway in human endothelial cells in pathophysiological conditions. J. Mol. Cell Cardiol. 37 (2), 515–523. 10.1016/j.yjmcc.2004.05.004 15276021

[B9] Bae S. W. Stuhlinger M. C. Yoo H. S. Yu K. H. Park H. K. Choi B. Y. (2005). Plasma asymmetric dimethylarginine concentrations in newly diagnosed patients with acute myocardial infarction or unstable angina pectoris during two weeks of medical treatment. Am. J. Cardiol. 95 (6), 729–733. 10.1016/j.amjcard.2004.11.023 15757598

[B10] Bagi Z. Erdei N. Papp Z. Edes I. Koller A. (2006). Up-regulation of vascular cyclooxygenase-2 in diabetes mellitus. Pharmacol. Rep. 58 Suppl, 52–56. 17332672

[B11] Barker D. J. Bagby S. P. Hanson M. A. (2006). Mechanisms of disease: in utero programming in the pathogenesis of hypertension. Nat. Clin. Pract. Nephrol. 2 (12), 700–707. 10.1038/ncpneph0344 17124527

[B12] Berkowitz D. E. White R. Li D. Minhas K. M. Cernetich A. Kim S. (2003). Arginase reciprocally regulates nitric oxide synthase activity and contributes to endothelial dysfunction in aging blood vessels. Circulation 108 (16), 2000–2006. 10.1161/01.CIR.0000092948.04444.C7 14517171

[B13] Bernstein R. D. Ochoa F. Y. Xu X. Forfia P. Shen W. Thompson C. II (1996). Function and production of nitric oxide in the coronary circulation of the conscious dog during exercise. Circ. Res. 79 (4), 840–848. 10.1161/01.res.79.4.840 8831509

[B14] Blum A. Hathaway L. Mincemoyer R. Schenke W. H. Kirby M. Csako G. (2000). Oral L-arginine in patients with coronary artery disease on medical management. Circulation 101 (18), 2160–2164. 10.1161/01.cir.101.18.2160 10801756

[B15] Bode-Boger S. M. Boger R. H. Creutzig A. Tsikas D. Gutzki F. M. Alexander K. (1994). L-arginine infusion decreases peripheral arterial resistance and inhibits platelet aggregation in healthy subjects. Clin. Sci. (Lond.) 87 (3), 303–310. 10.1042/cs0870303 7955906

[B16] Bode-Boger S. M. Scalera F. Ignarro L. J. (2007). The L-arginine paradox: Importance of the L-arginine/asymmetrical dimethylarginine ratio. Pharmacol. Ther. 114 (3), 295–306. 10.1016/j.pharmthera.2007.03.002 17482266

[B17] Boger R. H. Bode-Boger S. M. Brandes R. P. Phivthong-ngam L. Bohme M. Nafe R. (1997). Dietary L-arginine reduces the progression of atherosclerosis in cholesterol-fed rabbits: comparison with lovastatin. Circulation 96 (4), 1282–1290. 10.1161/01.cir.96.4.1282 9286960

[B18] Boger R. H. Maas R. Schulze F. Schwedhelm E. (2009). Asymmetric dimethylarginine (ADMA) as a prospective marker of cardiovascular disease and mortality–an update on patient populations with a wide range of cardiovascular risk. Pharmacol. Res. 60 (6), 481–487. 10.1016/j.phrs.2009.07.001 19596069

[B19] Boger R. H. (2004). Asymmetric dimethylarginine, an endogenous inhibitor of nitric oxide synthase, explains the “L-arginine paradox” and acts as a novel cardiovascular risk factor. J. Nutr. 134 (10 Suppl), 2842S–2847S. 10.1093/jn/134.10.2842S. discussion 2853S. 15465797

[B20] Boger R. H. (2005). Asymmetric dimethylarginine (ADMA) and cardiovascular disease: insights from prospective clinical trials. Vasc. Med. 10 Suppl 1, S19–S25. 10.1177/1358836X0501000104 16444865

[B21] Borgeraas H. Strand E. Ringdal Pedersen E. Dierkes J. Ueland P. M. Seifert R. (2012). Omega-3 Status and the Relationship between Plasma Asymmetric Dimethylarginine and Risk of Myocardial Infarction in Patients with Suspected Coronary Artery Disease. Cardiol. Res. Pract. 2012, 201742. 10.1155/2012/201742 23346455 PMC3549394

[B22] Bosmans J. M. Vrints C. J. Kockx M. M. Bult H. Cromheeke K. M. Herman A. G. (1999). Continuous perivascular L-arginine delivery increases total vessel area and reduces neointimal thickening after experimental balloon dilatation. Arterioscler. Thromb. Vasc. Biol. 19 (3), 767–776. 10.1161/01.atv.19.3.767 10073985

[B23] Broekhuizen L. N. Lemkes B. A. Mooij H. L. Meuwese M. C. Verberne H. Holleman F. (2010). Effect of sulodexide on endothelial glycocalyx and vascular permeability in patients with type 2 diabetes mellitus. Diabetologia 53 (12), 2646–2655. 10.1007/s00125-010-1910-x 20865240 PMC2974920

[B24] Cable D. G. Celotto A. C. Evora P. R. Schaff H. V. (2009). Asymmetric dimethylarginine endogenous inhibition of nitric oxide synthase causes differential vasculature effects. Med. Sci. Monit. 15 (9), BR248–BR253. 19721392

[B25] Ceremuzynski L. Chamiec T. Herbaczynska-Cedro K. (1997). Effect of supplemental oral L-arginine on exercise capacity in patients with stable angina pectoris. Am. J. Cardiol. 80 (3), 331–333. 10.1016/s0002-9149(97)00354-8 9264427

[B26] Cherry P. D. Furchgott R. F. Zawadzki J. V. Jothianandan D. (1982). Role of endothelial cells in relaxation of isolated arteries by bradykinin. Proc. Natl. Acad. Sci. U.S.A. 79 (6), 2106–2110. 10.1073/pnas.79.6.2106 6952258 PMC346132

[B27] Chester A. H. O’Neil G. S. Tadjkarimi S. Palmer R. M. Moncada S. Yacoub M. H. (1990). The role of nitric oxide in mediating endothelium dependent relaxations in the human epicardial coronary artery. Int. J. Cardiol. 29 (3), 305–309. 10.1016/0167-5273(90)90118-o 1704357

[B28] Chia S. Nagurney J. T. Brown D. F. Raffel O. C. Bamberg F. Senatore F. (2009). Association of leukocyte and neutrophil counts with infarct size, left ventricular function and outcomes after percutaneous coronary intervention for ST-elevation myocardial infarction. Am. J. Cardiol. 103 (3), 333–337. 10.1016/j.amjcard.2008.09.085 19166685

[B29] Clarkson P. Adams M. R. Powe A. J. Donald A. E. McCredie R. Robinson J. (1996). Oral L-arginine improves endothelium-dependent dilation in hypercholesterolemic young adults. J. Clin. Invest. 97 (8), 1989–1994. 10.1172/JCI118632 8621785 PMC507270

[B30] Cocks T. M. Angus J. A. Campbell J. H. Campbell G. R. (1985). Release and properties of endothelium-derived relaxing factor (EDRF) from endothelial cells in culture. J. Cell Physiol. 123 (3), 310–320. 10.1002/jcp.1041230304 3886674

[B31] Cooke J. P. Singer A. H. Tsao P. Zera P. Rowan R. A. Billingham M. E. (1992). Antiatherogenic effects of L-arginine in the hypercholesterolemic rabbit. J. Clin. Invest. 90 (3), 1168–1172. 10.1172/JCI115937 1522225 PMC329981

[B32] Cziraki A. Ajtay Z. Nemeth A. Lenkey Z. Sulyok E. Szabados S. (2011). Effects of coronary revascularization with or without cardiopulmonary bypass on plasma levels of asymmetric dimethylarginine. Coron. Artery Dis. 22 (4), 245–252. 10.1097/MCA.0b013e3283441d5c 21383621

[B33] Daiber A. Munzel T. Gori T. (2010). Organic nitrates and nitrate tolerance–state of the art and future developments. Adv. Pharmacol. 60, 177–227. 10.1016/B978-0-12-385061-4.00007-6 21081219

[B34] DeFronzo R. A. Ferrannini E. (1991). Insulin resistance. A multifaceted syndrome responsible for NIDDM, obesity, hypertension, dyslipidemia, and atherosclerotic cardiovascular disease. Diabetes Care 14 (3), 173–194. 10.2337/diacare.14.3.173 2044434

[B35] Dekker N. A. M. Veerhoek D. Koning N. J. van Leeuwen A. L. II Elbers P. W. G. van den Brom C. E. (2019). Postoperative microcirculatory perfusion and endothelial glycocalyx shedding following cardiac surgery with cardiopulmonary bypass. Anaesthesia 74 (5), 609–618. 10.1111/anae.14577 30687934 PMC6590376

[B36] Di Luozzo G. Bhargava J. Powell R. J. (2000). Vascular smooth muscle cell effect on endothelial cell endothelin-1 production. J. Vasc. Surg. 31 (4), 781–789. 10.1067/mva.2000.103788 10753286

[B37] Dong J. Y. Qin L. Q. Zhang Z. Zhao Y. Wang J. Arigoni F. (2011). Effect of oral L-arginine supplementation on blood pressure: a meta-analysis of randomized, double-blind, placebo-controlled trials. Am. Heart J. 162 (6), 959–965. 10.1016/j.ahj.2011.09.012 22137067

[B38] Dornyei G. Monos E. Kaley G. Koller A. (2000). Regular exercise enhances blood pressure lowering effect of acetylcholine by increased contribution of nitric oxide. Acta Physiol. Hung 87 (2), 127–138. 11205960

[B39] Dragovich M. A. Chester D. Fu B. M. Wu C. Xu Y. Goligorsky M. S. (2016). Mechanotransduction of the endothelial glycocalyx mediates nitric oxide production through activation of TRP channels. Am. J. Physiol. Cell Physiol. 311 (6), C846–C853. 10.1152/ajpcell.00288.2015 27681180

[B40] Dubois-Rande J. L. Zelinsky R. Roudot F. Chabrier P. E. Castaigne A. Geschwind H. (1992). Effects of infusion of L-arginine into the left anterior descending coronary artery on acetylcholine-induced vasoconstriction of human atheromatous coronary arteries. Am. J. Cardiol. 70 (15), 1269–1275. 10.1016/0002-9149(92)90760-v 1442577

[B41] Duffy S. J. Castle S. F. Harper R. W. Meredith I. T. (1999). Contribution of vasodilator prostanoids and nitric oxide to resting flow, metabolic vasodilation, and flow-mediated dilation in human coronary circulation. Circulation 100 (19), 1951–1957. 10.1161/01.cir.100.19.1951 10556220

[B42] Durante W. Johnson F. K. Johnson R. A. (2007). Arginase: a critical regulator of nitric oxide synthesis and vascular function. Clin. Exp. Pharmacol. Physiol. 34 (9), 906–911. 10.1111/j.1440-1681.2007.04638.x 17645639 PMC1955221

[B43] Egashira K. Hirooka Y. Kuga T. Mohri M. Takeshita A. (1996a). Effects of L-arginine supplementation on endothelium-dependent coronary vasodilation in patients with angina pectoris and normal coronary arteriograms. Circulation 94 (2), 130–134. 10.1161/01.cir.94.2.130 8674170

[B44] Egashira K. Katsuda Y. Mohri M. Kuga T. Tagawa T. Kubota T. (1996b). Role of endothelium-derived nitric oxide in coronary vasodilatation induced by pacing tachycardia in humans. Circ. Res. 79 (2), 331–335. 10.1161/01.res.79.2.331 8756012

[B45] El-Kirsh A. A. Abd El-Wahab H. M. Abd-Ellah Sayed H. F. (2011). The effect of L-arginine or L-citrulline supplementation on biochemical parameters and the vascular aortic wall in high-fat and high-cholesterol-fed rats. Cell Biochem. Funct. 29 (5), 414–428. 10.1002/cbf.1766 21638297

[B46] Feng Q. Lu X. Fortin A. J. Pettersson A. Hedner T. Kline R. L. (1998). Elevation of an endogenous inhibitor of nitric oxide synthesis in experimental congestive heart failure. Cardiovasc. Res. 37 (3), 667–675. 10.1016/s0008-6363(97)00242-3 9659450

[B47] Fujita H. Takeda K. Miki S. Harada S. Hatta T. Uchida A. (2000). Effect of L-arginine on endothelium-dependent coronary vasodilatory reserve in spontaneously hypertensive rats. Curr. Ther. Res. Clin. Exp. 61 (10), 680–689. 10.1016/S0011-393X(00)80048-X

[B48] Fukuto J. M. Wood K. S. Byrns R. E. Ignarro L. J. (1990). NG-amino-L-arginine: a new potent antagonist of L-arginine-mediated endothelium-dependent relaxation. Biochem. Biophys. Res. Commun. 168 (2), 458–465. 10.1016/0006-291x(90)92343-x 2159292

[B49] Furchgott R. F. Zawadzki J. V. (1980). The obligatory role of endothelial cells in the relaxation of arterial smooth muscle by acetylcholine. Nature 288 (5789), 373–376. 10.1038/288373a0 6253831

[B50] Gao X. Zhang H. Belmadani S. Wu J. Xu X. Elford H. (2008). Role of TNF-alpha-induced reactive oxygen species in endothelial dysfunction during reperfusion injury. Am. J. Physiol. Heart Circ. Physiol. 295 (6), H2242–H2249. 10.1152/ajpheart.00587.2008 18849334 PMC2614540

[B51] Gold M. E. Wood K. S. Byrns R. E. Buga G. M. Ignarro L. J. (1990). L-arginine-dependent vascular smooth muscle relaxation and cGMP formation. Am. J. Physiol. 259 (6 Pt 2), H1813–H1821. 10.1152/ajpheart.1990.259.6.H1813 2175566

[B52] Gori T. Daiber A. (2009). Non-hemodynamic effects of organic nitrates and the distinctive characteristics of pentaerithrityl tetranitrate. Am. J. Cardiovasc. Drugs 9 (1), 7–15. 10.1007/BF03256591 19178128

[B53] Habib G. B. Heibig J. Forman S. A. Brown B. G. Roberts R. Terrin M. L. (1991). Influence of coronary collateral vessels on myocardial infarct size in humans. Results of phase I thrombolysis in myocardial infarction (TIMI) trial. The TIMI Investigators. Circulation 83 (3), 739–746. 10.1161/01.cir.83.3.739 1900223

[B54] Hadi H. A. Carr C. S. Al Suwaidi J. (2005). Endothelial dysfunction: cardiovascular risk factors, therapy, and outcome. Vasc. Health Risk Manag. 1 (3), 183–198. 17319104 PMC1993955

[B55] Hein T. W. Zhang C. Wang W. Chang C. II Thengchaisri N. Kuo L. (2003). Ischemia-reperfusion selectively impairs nitric oxide-mediated dilation in coronary arterioles: counteracting role of arginase. FASEB J. 17 (15), 2328–2330. 10.1096/fj.03-0115fje 14563685

[B56] Higashi Y. Oshima T. Ono N. Hiraga H. Yoshimura M. Watanabe M. (1995). Intravenous administration of L-arginine inhibits angiotensin-converting enzyme in humans. J. Clin. Endocrinol. Metab. 80 (7), 2198–2202. 10.1210/jcem.80.7.7608279 7608279

[B57] Hishikawa K. Nakaki T. Tsuda M. Esumi H. Ohshima H. Suzuki H. (1992). Effect of systemic L-arginine administration on hemodynamics and nitric oxide release in man. Jpn. Heart J. 33 (1), 41–48. 10.1536/ihj.33.41 1315399

[B58] Huang A. Sun D. Shesely E. G. Levee E. M. Koller A. Kaley G. (2002). Neuronal NOS-dependent dilation to flow in coronary arteries of male eNOS-KO mice. Am. J. Physiol. Heart Circ. Physiol. 282 (2), H429–H436. 10.1152/ajpheart.00501.2001 11788389

[B59] Ignarro L. J. Napoli C. Loscalzo J. (2002). Nitric oxide donors and cardiovascular agents modulating the bioactivity of nitric oxide: an overview. Circ. Res. 90 (1), 21–28. 10.1161/hh0102.102330 11786514

[B60] Investigators G. A. (1993). The effects of tissue plasminogen activator, streptokinase, or both on coronary-artery patency, ventricular function, and survival after acute myocardial infarction. N Engl. J. Med. 329 (22), 1615–1622. 10.1056/NEJM199311253292204 8232430

[B61] Janus A. Szahidewicz-Krupska E. Mazur G. Doroszko A. (2016). Insulin Resistance and Endothelial Dysfunction Constitute a Common Therapeutic Target in Cardiometabolic Disorders. Mediators Inflammation 2016, 3634948. 10.1155/2016/3634948 PMC4931075 27413253

[B62] Jenkinson C. P. Grody W. W. Cederbaum S. D. (1996). Comparative properties of arginases. Comp. Biochem. Physiol. B Biochem. Mol. Biol. 114 (1), 107–132. 10.1016/0305-0491(95)02138-8 8759304

[B63] Ji H. Li H. Martasek P. Roman L. J. Poulos T. L. Silverman R. B. (2009). Discovery of highly potent and selective inhibitors of neuronal nitric oxide synthase by fragment hopping. J. Med. Chem. 52 (3), 779–797. 10.1021/jm801220a 19125620 PMC2664101

[B64] Kaley G. Koller A. Rodenburg J. M. Messina E. J. Wolin M. S. (1992). Regulation of arteriolar tone and responses via L-arginine pathway in skeletal muscle. Am. J. Physiol. 262 (4 Pt 2), H987–H992. 10.1152/ajpheart.1992.262.4.H987 1566917

[B65] Kawamoto O. Michiue T. Ishikawa T. Maeda H. (2013). Comprehensive evaluation of pericardial biochemical markers in death investigation. Forensic Sci. Int. 224 (1-3), 73–79. 10.1016/j.forsciint.2012.10.036 23196195

[B66] Khalaf D. Krüger M. Wehland M. Infanger M. Grimm D. (2019). The Effects of Oral l-Arginine and l-Citrulline Supplementation on Blood Pressure. Nutrients 11 (7), 1679. 10.3390/nu11071679 PMC6683098 31336573

[B67] Khan S. G. Melikian N. Shabeeh H. Cabaco A. R. Martin K. Khan F. (2017). The human coronary vasodilatory response to acute mental stress is mediated by neuronal nitric oxide synthase. Am. J. Physiol. Heart Circ. Physiol. 313 (3), H578–H583. 10.1152/ajpheart.00745.2016 28646032 PMC5625168

[B68] Kiss T. Tarantini S. Csipo T. Balasubramanian P. Nyul-Toth A. Yabluchanskiy A. (2020). Circulating anti-geronic factors from heterochonic parabionts promote vascular rejuvenation in aged mice: transcriptional footprint of mitochondrial protection, attenuation of oxidative stress, and rescue of endothelial function by young blood. Geroscience 42 (2), 727–748. 10.1007/s11357-020-00180-6 32172434 PMC7205954

[B69] Kocaman S. A. Sahinarslan A. Biberoglu G. Hasanoglu A. Akyel A. Timurkaynak T. (2008). Asymmetric dimethylarginine and coronary collateral vessel development. Coron. Artery Dis. 19 (7), 469–474. 10.1097/MCA.0b013e328311d32b 18923242

[B70] Koller A. Messina E. J. Wolin M. S. Kaley G. (1989). Effects of endothelial impairment on arteriolar dilator responses in vivo. Am. J. Physiol. 257 (5 Pt 2), H1485–H1489. 10.1152/ajpheart.1989.257.5.H1485 2589504

[B71] Koller A. Sun D. Messina E. J. Kaley G. (1993). L-arginine analogues blunt prostaglandin-related dilation of arterioles. Am. J. Physiol. 264 (4 Pt 2), H1194–H1199. 10.1152/ajpheart.1993.264.4.H1194 8476097

[B72] Koller A. Sun D. Huang A. Kaley G. (1994). Corelease of nitric oxide and prostaglandins mediates flow-dependent dilation of rat gracilis muscle arterioles. Am. J. Physiol. 267 (1 Pt 2), H326–H332. 10.1152/ajpheart.1994.267.1.H326 8048598

[B73] Koller A. Huang A. Sun D. Kaley G. (1995). Exercise training augments flow-dependent dilation in rat skeletal muscle arterioles. Role of endothelial nitric oxide and prostaglandins. Circ. Res. 76 (4), 544–550. 10.1161/01.res.76.4.544 7534658

[B74] Kovamees O. Shemyakin A. Pernow J. (2014). Effect of arginase inhibition on ischemia-reperfusion injury in patients with coronary artery disease with and without diabetes mellitus. PloS One 9 (7), e103260. 10.1371/journal.pone.0103260 25072937 PMC4114552

[B75] Kruszelnicka O. Surdacki A. Golay A. (2013). Differential associations of angiographic extent and severity of coronary artery disease with asymmetric dimethylarginine but not insulin resistance in non-diabetic men with stable angina: a cross-sectional study. Cardiovasc. Diabetol. 12, 145. 10.1186/1475-2840-12-145 24103320 PMC3852014

[B76] Ku D. D. (1982). Coronary vascular reactivity after acute myocardial ischemia. Science 218 (4572), 576–578. 10.1126/science.7123259 7123259

[B77] Kuo L. Hein T. W. (2013). Vasomotor regulation of coronary microcirculation by oxidative stress: role of arginase. Front. Immunol. 4, 237. 10.3389/fimmu.2013.00237 23966996 PMC3746455

[B78] Kusche-Vihrog K. Jeggle P. Oberleithner H. (2014). The role of ENaC in vascular endothelium. Pflugers Arch. 466 (5), 851–859. 10.1007/s00424-013-1356-3 24046153

[B79] Laughlin M. H. Bowles D. K. Duncker D. J. (2012). The coronary circulation in exercise training. Am. J. Physiol. Heart Circ. Physiol. 302 (1), H10–H23. 10.1152/ajpheart.00574.2011 21984538 PMC3334245

[B80] Lefroy D. C. Crake T. Uren N. G. Davies G. J. Maseri A. (1993). Effect of inhibition of nitric oxide synthesis on epicardial coronary artery caliber and coronary blood flow in humans. Circulation 88 (1), 43–54. 10.1161/01.cir.88.1.43 8319355

[B81] Lerman A. Burnett J. C. Jr. Higano S. T. McKinley L. J. Holmes D. R. Jr. (1998). Long-term L-arginine supplementation improves small-vessel coronary endothelial function in humans. Circulation 97 (21), 2123–2128. 10.1161/01.cir.97.21.2123 9626172

[B82] Li H. Meininger C. J. Hawker J. R. Jr. Haynes T. E. Kepka-Lenhart D. Mistry S. K. (2001). Regulatory role of arginase I and II in nitric oxide, polyamine, and proline syntheses in endothelial cells. Am. J. Physiol. Endocrinol. Metab. 280 (1), E75–E82. 10.1152/ajpendo.2001.280.1.E75 11120661

[B83] Loscalzo J. (2013). The identification of nitric oxide as endothelium-derived relaxing factor. Circ. Res. 113 (2), 100–103. 10.1161/CIRCRESAHA.113.301577 23833290 PMC3711180

[B84] Lu T. M. Ding Y. A. Charng M. J. Lin S. J. (2003a). Asymmetrical dimethylarginine: a novel risk factor for coronary artery disease. Clin. Cardiol. 26 (10), 458–464. 10.1002/clc.4960261006 14579916 PMC6654648

[B85] Lu T. M. Ding Y. A. Lin S. J. Lee W. S. Tai H. C. (2003b). Plasma levels of asymmetrical dimethylarginine and adverse cardiovascular events after percutaneous coronary intervention. Eur. Heart J. 24 (21), 1912–1919. 10.1016/j.ehj.2003.08.013 14585249

[B86] Lundberg J. O. Weitzberg E. Gladwin M. T. (2008). The nitrate-nitrite-nitric oxide pathway in physiology and therapeutics. Nat. Rev. Drug Discovery 7 (2), 156–167. 10.1038/nrd2466 18167491

[B87] Luque Contreras D. Vargas Robles H. Romo E. Rios A. Escalante B. (2006). The role of nitric oxide in the post-ischemic revascularization process. Pharmacol. Ther. 112 (2), 553–563. 10.1016/j.pharmthera.2006.05.003 16950515

[B88] Luscher T. F. Yang Z. Tschudi M. von Segesser L. Stulz P. Boulanger C. (1990). Interaction between endothelin-1 and endothelium-derived relaxing factor in human arteries and veins. Circ. Res. 66 (4), 1088–1094. 10.1161/01.res.66.4.1088 2180587

[B89] Mangiacapra F. Conte M. Demartini C. Muller O. Delrue L. Dierickx K. (2016). Relationship of asymmetric dimethylarginine (ADMA) with extent and functional severity of coronary atherosclerosis. Int. J. Cardiol. 220, 629–633. 10.1016/j.ijcard.2016.06.254 27391005

[B90] Meinitzer A. Seelhorst U. Wellnitz B. Halwachs-Baumann G. Boehm B. O. Winkelmann B. R. (2007). Asymmetrical dimethylarginine independently predicts total and cardiovascular mortality in individuals with angiographic coronary artery disease (the Ludwigshafen Risk and Cardiovascular Health study). Clin. Chem. 53 (2), 273–283. 10.1373/clinchem.2006.076711 17185364

[B91] Moncada S. Higgs A. (1993). The L-arginine-nitric oxide pathway. N Engl. J. Med. 329 (27), 2002–2012. 10.1056/NEJM199312303292706 7504210

[B92] Moncada S. Higgs E. A. (2006). The discovery of nitric oxide and its role in vascular biology. Br. J. Pharmacol. 147 Suppl 1, S193–S201. 10.1038/sj.bjp.0706458 16402104 PMC1760731

[B93] Morita M. Hayashi T. Ochiai M. Maeda M. Yamaguchi T. Ina K. (2014). Oral supplementation with a combination of L-citrulline and L-arginine rapidly increases plasma L-arginine concentration and enhances NO bioavailability. Biochem. Biophys. Res. Commun. 454 (1), 53–57. 10.1016/j.bbrc.2014.10.029 25445598

[B94] Mulivor A. W. Lipowsky H. H. (2004). Inflammation- and ischemia-induced shedding of venular glycocalyx. Am. J. Physiol. Heart Circ. Physiol. 286 (5), H1672–H1680. 10.1152/ajpheart.00832.2003 14704229

[B95] Muniyappa R. Sowers J. R. (2013). Role of insulin resistance in endothelial dysfunction. Rev. Endocr. Metab. Disord. 14 (1), 5–12. 10.1007/s11154-012-9229-1 23306778 PMC3594115

[B96] Muniyappa R. Montagnani M. Koh K. K. Quon M. J. (2007). Cardiovascular actions of insulin. Endocr. Rev. 28 (5), 463–491. 10.1210/er.2007-0006 17525361

[B97] Murohara T. Asahara T. Silver M. Bauters C. Masuda H. Kalka C. (1998). Nitric oxide synthase modulates angiogenesis in response to tissue ischemia. J. Clin. Invest. 101 (11), 2567–2578. 10.1172/JCI1560 9616228 PMC508846

[B98] Nemeth Z. Cziraki A. Szabados S. Biri B. Keki S. Koller A. (2015a). Elevated Levels of Asymmetric Dimethylarginine (ADMA) in the Pericardial Fluid of Cardiac Patients Correlate with Cardiac Hypertrophy. PloS One 10 (8), e0135498. 10.1371/journal.pone.0135498 26313940 PMC4551682

[B99] Nemeth Z. Cziraki A. Szabados S. Horvath I. Koller A. (2015b). Pericardial fluid of cardiac patients elicits arterial constriction: role of endothelin-1. Can. J. Physiol. Pharmacol. 93 (9), 779–785. 10.1139/cjpp-2015-0030 26322806

[B100] Neri I. Monari F. Sgarbi L. Berardi A. Masellis G. Facchinetti F. (2010). L-arginine supplementation in women with chronic hypertension: impact on blood pressure and maternal and neonatal complications. J. Matern. Fetal Neonatal Med. 23 (12), 1456–1460. 10.3109/14767051003677962 20958228

[B101] Nijveldt R. J. Teerlink T. Siroen M. P. van Lambalgen A. A. Rauwerda J. A. van Leeuwen P. A. (2003a). The liver is an important organ in the metabolism of asymmetrical dimethylarginine (ADMA). Clin. Nutr. 22 (1), 17–22. 10.1054/clnu.2002.0612 12553945

[B102] Nijveldt R. J. Teerlink T. van Guldener C. Prins H. A. van Lambalgen A. A. Stehouwer C. D. (2003b). Handling of asymmetrical dimethylarginine and symmetrical dimethylarginine by the rat kidney under basal conditions and during endotoxaemia. Nephrol. Dial. Transplant. 18 (12), 2542–2550. 10.1093/ndt/gfg452 14605276

[B103] Nishikawa Y. Ogawa S. (1997). Importance of nitric oxide in the coronary artery at rest and during pacing in humans. J. Am. Coll. Cardiol. 29 (1), 85–92. 10.1016/s0735-1097(96)00429-9 8996299

[B104] Ogawa T. Kimoto M. Sasaoka K. (1989). Purification and properties of a new enzyme, NG,NG-dimethylarginine dimethylaminohydrolase, from rat kidney. J. Biol. Chem. 264 (17), 10205–10209. 2722865

[B105] Olde Engberink R. H. Rorije N. M. Homan van der Heide J. J. van den Born B. J. Vogt L. (2015). Role of the vascular wall in sodium homeostasis and salt sensitivity. J. Am. Soc. Nephrol. 26 (4), 777–783. 10.1681/ASN.2014050430 25294232 PMC4378110

[B106] Padro T. Manfrini O. Bugiardini R. Canty J. Cenko E. De Luca G. (2020). ESC Working Group on Coronary Pathophysiology and Microcirculation position paper on ‘coronary microvascular dysfunction in cardiovascular disease’. Cardiovasc. Res. 116 (4), 741–755. 10.1093/cvr/cvaa003 32034397 PMC7825482

[B107] Palm F. Onozato M. L. Luo Z. Wilcox C. S. (2007). Dimethylarginine dimethylaminohydrolase (DDAH): expression, regulation, and function in the cardiovascular and renal systems. Am. J. Physiol. Heart Circ. Physiol. 293 (6), H3227–H3245. 10.1152/ajpheart.00998.2007 17933965

[B108] Palmer R. M. Ashton D. S. Moncada S. (1988a). Vascular endothelial cells synthesize nitric oxide from L-arginine. Nature 333 (6174), 664–666. 10.1038/333664a0 3131684

[B109] Palmer R. M. Rees D. D. Ashton D. S. Moncada S. (1988b). L-arginine is the physiological precursor for the formation of nitric oxide in endothelium-dependent relaxation. Biochem. Biophys. Res. Commun. 153 (3), 1251–1256. 10.1016/s0006-291x(88)81362-7 3390182

[B110] Parikh R. V. Khush K. K. Luikart H. Pargaonkar V. S. Kobayashi Y. Lee J. H. (2017). Impact of Asymmetric Dimethylarginine on Coronary Physiology Early After Heart Transplantation. Am. J. Cardiol. 120 (6), 1020–1025. 10.1016/j.amjcard.2017.06.036 28754566

[B111] Pope A. J. Karrupiah K. Kearns P. N. Xia Y. Cardounel A. J. (2009). Role of dimethylarginine dimethylaminohydrolases in the regulation of endothelial nitric oxide production. J. Biol. Chem. 284 (51), 35338–35347. 10.1074/jbc.M109.037036 19820234 PMC2790963

[B112] Quyyumi A. A. Dakak N. Andrews N. P. Gilligan D. M. Panza J. A. Cannon, 3rd R. O. (1995a). Contribution of nitric oxide to metabolic coronary vasodilation in the human heart. Circulation 92 (3), 320–326. 10.1161/01.cir.92.3.320 7634444

[B113] Quyyumi A. A. Dakak N. Andrews N. P. Husain S. Arora S. Gilligan D. M. (1995b). Nitric oxide activity in the human coronary circulation. Impact of risk factors for coronary atherosclerosis. J. Clin. Invest. 95 (4), 1747–1755. 10.1172/JCI117852 7706483 PMC295695

[B114] Quyyumi A. A. Dakak N. Diodati J. G. Gilligan D. M. Panza J. A. Cannon, 3rd R. O. (1997a). Effect of L-arginine on human coronary endothelium-dependent and physiologic vasodilation. J. Am. Coll. Cardiol. 30 (5), 1220–1227. 10.1016/s0735-1097(97)00279-9 9350919

[B115] Quyyumi A. A. Mulcahy D. Andrews N. P. Husain S. Panza J. A. Cannon, 3rd R. O. (1997b). Coronary vascular nitric oxide activity in hypertension and hypercholesterolemia. Comparison of acetylcholine and substance P. Circulation 95 (1), 104–110. 10.1161/01.cir.95.1.104 8994424

[B116] Quyyumi A. A. (1998). Does acute improvement of endothelial dysfunction in coronary artery disease improve myocardial ischemia? A double-blind comparison of parenteral D- and L-arginine. J. Am. Coll. Cardiol. 32 (4), 904–911. 10.1016/s0735-1097(98)00323-4 9768710

[B117] Rees D. Ben-Ishay D. Moncada S. (1996). Nitric oxide and the regulation of blood pressure in the hypertension-prone and hypertension-resistant Sabra rat. Hypertension 28 (3), 367–371. 10.1161/01.hyp.28.3.367 8794818

[B118] Rehm M. Bruegger D. Christ F. Conzen P. Thiel M. Jacob M. (2007). Shedding of the endothelial glycocalyx in patients undergoing major vascular surgery with global and regional ischemia. Circulation 116 (17), 1896–1906. 10.1161/CIRCULATIONAHA.106.684852 17923576

[B119] Reitsma S. Slaaf D. W. Vink H. van Zandvoort M. A. oude Egbrink M. G. (2007). The endothelial glycocalyx: composition, functions, and visualization. Pflugers Arch. 454 (3), 345–359. 10.1007/s00424-007-0212-8 17256154 PMC1915585

[B120] Rochette L. Lorin J. Zeller M. Guilland J. C. Lorgis L. Cottin Y. (2013). Nitric oxide synthase inhibition and oxidative stress in cardiovascular diseases: possible therapeutic targets? Pharmacol. Ther. 140 (3), 239–257. 10.1016/j.pharmthera.2013.07.004 23859953

[B121] Sankaralingam S. Xu H. Davidge S. T. (2010). Arginase contributes to endothelial cell oxidative stress in response to plasma from women with preeclampsia. Cardiovasc. Res. 85 (1), 194–203. 10.1093/cvr/cvp277 19684035

[B122] Scalera F. Martens-Lobenhoffer J. Tager M. Bukowska A. Lendeckel U. Bode-Boger S. M. (2006). Effect of L-arginine on asymmetric dimethylarginine (ADMA) or homocysteine-accelerated endothelial cell aging. Biochem. Biophys. Res. Commun. 345 (3), 1075–1082. 10.1016/j.bbrc.2006.05.015 16713997

[B123] Schneider J. Y. Rothmann S. Schroder F. Langen J. Lucke T. Mariotti F. (2015). Effects of chronic oral L-arginine administration on the L-arginine/NO pathway in patients with peripheral arterial occlusive disease or coronary artery disease: L-Arginine prevents renal loss of nitrite, the major NO reservoir. Amino Acids 47 (9), 1961–1974. 10.1007/s00726-015-2031-0 26123989

[B124] Schuhmacher S. Schulz E. Oelze M. Konig A. Roegler C. Lange K. (2009). A new class of organic nitrates: investigations on bioactivation, tolerance and cross-tolerance phenomena. Br. J. Pharmacol. 158 (2), 510–520. 10.1111/j.1476-5381.2009.00303.x 19563531 PMC2757691

[B125] Schulze F. Lenzen H. Hanefeld C. Bartling A. Osterziel K. J. Goudeva L. (2006). Asymmetric dimethylarginine is an independent risk factor for coronary heart disease: results from the multicenter Coronary Artery Risk Determination investigating the Influence of ADMA Concentration (CARDIAC) study. Am. Heart J. 152 (3), 493 e491–498. 10.1016/j.ahj.2006.06.005 16923419

[B126] Searles C. D. (2002). The nitric oxide pathway and oxidative stress in heart failure. Congest. Heart Fail 8 (3), 142–147, 155. 10.1111/j.1527-5299.2002.00715.x 12045382

[B127] Seddon M. Melikian N. Dworakowski R. Shabeeh H. Jiang B. Byrne J. (2009). Effects of neuronal nitric oxide synthase on human coronary artery diameter and blood flow in vivo. Circulation 119 (20), 2656–2662. 10.1161/CIRCULATIONAHA.108.822205 19433760

[B128] Seljeflot I. Nilsson B. B. Westheim A. S. Bratseth V. Arnesen H. (2011). “The L-arginine-asymmetric dimethylarginine ratio is strongly related to the severity of chronic heart failure. No effects of exercise training. J. Card. Fail 17 (2), 135–142. 10.1016/j.cardfail.2010.09.003 21300303

[B129] Shabeeh H. Melikian N. Dworakowski R. Casadei B. Chowienczyk P. Shah A. M. (2013). Differential role of endothelial versus neuronal nitric oxide synthase in the regulation of coronary blood flow during pacing-induced increases in cardiac workload. Am. J. Physiol. Heart Circ. Physiol. 304 (9), H1277–H1282. 10.1152/ajpheart.00927.2012 23479261 PMC3652090

[B130] Shemyakin A. Kovamees O. Rafnsson A. Bohm F. Svenarud P. Settergren M. (2012). Arginase inhibition improves endothelial function in patients with coronary artery disease and type 2 diabetes mellitus. Circulation 126 (25), 2943–2950. 10.1161/CIRCULATIONAHA.112.140335 23183942

[B131] Stone G. W. Brodie B. R. Griffin J. J. Morice M. C. Costantini C. St Goar F. G. (1998). Prospective, multicenter study of the safety and feasibility of primary stenting in acute myocardial infarction: in-hospital and 30-day results of the PAMI stent pilot trial. Primary Angioplasty in Myocardial Infarction Stent Pilot Trial Investigators. J. Am. Coll. Cardiol. 31 (1), 23–30. 10.1016/s0735-1097(97)00439-7 9426013

[B132] Suda O. Tsutsui M. Morishita T. Tasaki H. Ueno S. Nakata S. (2004). Asymmetric dimethylarginine produces vascular lesions in endothelial nitric oxide synthase-deficient mice: involvement of renin-angiotensin system and oxidative stress. Arterioscler. Thromb. Vasc. Biol. 24 (9), 1682–1688. 10.1161/01.ATV.0000136656.26019.6e 15217805

[B133] Sun D. Messina E. J. Koller A. Wolin M. S. Kaley G. (1992). Endothelium-dependent dilation to L-arginine in isolated rat skeletal muscle arterioles. Am. J. Physiol. 262 (4 Pt 2), H1211–H1216. 10.1152/ajpheart.1992.262.4.H1211 1566902

[B134] Suzuki T. Hayase M. Hibi K. Hosokawa H. Yokoya K. Fitzgerald P. J. (2002). Effect of local delivery of L-arginine on in-stent restenosis in humans. Am. J. Cardiol. 89 (4), 363–367. 10.1016/s0002-9149(01)02252-4 11835911

[B135] Szerafin T. Erdei N. Fulop T. Pasztor E. T. Edes I. Koller A. (2006). Increased cyclooxygenase-2 expression and prostaglandin-mediated dilation in coronary arterioles of patients with diabetes mellitus. Circ. Res. 99 (5), e12–e17. 10.1161/01.RES.0000241051.83067.62 16917094

[B136] Szokodi I. Horkay F. Merkely B. Solti F. Geller L. Kiss P. (1998). Intrapericardial infusion of endothelin-1 induces ventricular arrhythmias in dogs. Cardiovasc. Res. 38 (2), 356–364. 10.1016/s0008-6363(98)00018-2 9709396

[B137] Tangphao O. Chalon S. Coulston A. M. Moreno H. Jr. Chan J. R. Cooke J. P. (1999). L-arginine and nitric oxide-related compounds in plasma: comparison of normal and arginine-free diets in a 24-h crossover study. Vasc. Med. 4 (1), 27–32. 10.1177/1358836X9900400105 10355867

[B138] Thum T. Tsikas D. Stein S. Schultheiss M. Eigenthaler M. Anker S. D. (2005). Suppression of endothelial progenitor cells in human coronary artery disease by the endogenous nitric oxide synthase inhibitor asymmetric dimethylarginine. J. Am. Coll. Cardiol. 46 (9), 1693–1701. 10.1016/j.jacc.2005.04.066 16256870

[B139] Toda N. Okamura T. (2003). The pharmacology of nitric oxide in the peripheral nervous system of blood vessels. Pharmacol. Rev. 55 (2), 271–324. 10.1124/pr.55.2.3 12773630

[B140] Toda N. Toda H. (2011). Coronary hemodynamic regulation by nitric oxide in experimental animals: recent advances. Eur. J. Pharmacol. 667 (1-3), 41–49. 10.1016/j.ejphar.2011.06.028 21741964

[B141] Toth J. Racz A. Kaminski P. M. Wolin M. S. Bagi Z. Koller A. (2007). Asymmetrical dimethylarginine inhibits shear stress-induced nitric oxide release and dilation and elicits superoxide-mediated increase in arteriolar tone. Hypertension 49 (3), 563–568. 10.1161/01.HYP.0000256764.86208.3d 17242303

[B142] Tousoulis D. Tentolouris C. Crake T. Toutouzas P. Davies G. (1997). Basal and flow-mediated nitric oxide production by atheromatous coronary arteries. J. Am. Coll. Cardiol. 29 (6), 1256–1262. 10.1016/s0735-1097(97)00046-6 9137221

[B143] Tousoulis D. Tentolouris C. Crake T. Katsimaglis G. Stefanadis C. Toutouzas P. (1999). Effects of L- and D-arginine on the basal tone of human diseased coronary arteries and their responses to substance P. Heart 81 (5), 505–511. 10.1136/hrt.81.5.505 10212169 PMC1729035

[B144] Tousoulis D. Antoniades C. Tentolouris C. Goumas G. Stefanadis C. Toutouzas P. (2002). L-arginine in cardiovascular disease: dream or reality? Vasc. Med. 7 (3), 203–211. 10.1191/1358863x02vm434ra 12553744

[B145] Tousoulis D. Boger R. H. Antoniades C. Siasos G. Stefanadi E. Stefanadis C. (2007). Mechanisms of disease: L-arginine in coronary atherosclerosis–a clinical perspective. Nat. Clin. Pract. Cardiovasc. Med. 4 (5), 274–283. 10.1038/ncpcardio0878 17457351

[B146] Tousoulis D. Georgakis M. K. Oikonomou E. Papageorgiou N. Zaromitidou M. Latsios G. (2015). Asymmetric Dimethylarginine: Clinical Significance and Novel Therapeutic Approaches. Curr. Med. Chem. 22 (24), 2871–2901. 10.2174/0929867322666150625095046 26112145

[B147] Trochu J. N. Mital S. Zhang X. Xu X. Ochoa M. Liao J. K. (2003). Preservation of NO production by statins in the treatment of heart failure. Cardiovasc. Res. 60 (2), 250–258. 10.1016/j.cardiores.2003.08.003 14613854 PMC2653218

[B148] Udvardy M. Posan E. Palatka K. Altorjay I. Harsfalvi J. (1997). Effect of L-arginine on in vitro plasmin-generation and fibrinogenolysis. Thromb. Res. 87 (1), 75–82. 10.1016/s0049-3848(97)00106-0 9253802

[B149] Ueda S. Kato S. Matsuoka H. Kimoto M. Okuda S. Morimatsu M. (2003). Regulation of cytokine-induced nitric oxide synthesis by asymmetric dimethylarginine: role of dimethylarginine dimethylaminohydrolase. Circ. Res. 92 (2), 226–233. 10.1161/01.res.0000052990.68216.ef 12574151

[B150] Ungvari Z. Sun D. Huang A. Kaley G. Koller A. (2001). Role of endothelial [Ca2+]i in activation of eNOS in pressurized arterioles by agonists and wall shear stress. Am. J. Physiol. Heart Circ. Physiol. 281 (2), H606–H612. 10.1152/ajpheart.2001.281.2.H606 11454563

[B151] Ungvari Z. Csiszar A. Bagi Z. Koller A. (2002). Impaired nitric oxide-mediated flow-induced coronary dilation in hyperhomocysteinemia: morphological and functional evidence for increased peroxynitrite formation. Am. J. Pathol. 161 (1), 145–153. 10.1016/S0002-9440(10)64166-1 12107099 PMC1850707

[B152] Ungvari Z. Tarantini S. Sorond F. Merkely B. Csiszar A. (2020). Mechanisms of Vascular Aging, A Geroscience Perspective: JACC Focus Seminar. J. Am. Coll. Cardiol. 75 (8), 931–941. 10.1016/j.jacc.2019.11.061 32130929 PMC8559983

[B153] Vaccarino V. Badimon L. Corti R. de Wit C. Dorobantu M. Manfrini O. (2013). Presentation, management, and outcomes of ischaemic heart disease in women. Nat. Rev. Cardiol. 10 (9), 508–518. 10.1038/nrcardio.2013.93 23817188 PMC10878732

[B154] Vallance P. Leiper J. (2004). Cardiovascular biology of the asymmetric dimethylarginine:dimethylarginine dimethylaminohydrolase pathway. Arterioscler. Thromb. Vasc. Biol. 24 (6), 1023–1030. 10.1161/01.ATV.0000128897.54893.26 15105281

[B155] Vallance P. Leone A. Calver A. Collier J. Moncada S. (1992). Accumulation of an endogenous inhibitor of nitric oxide synthesis in chronic renal failure. Lancet 339 (8793), 572–575. 10.1016/0140-6736(92)90865-z 1347093

[B156] van den Berg B. M. Vink H. Spaan J. A. (2003). The endothelial glycocalyx protects against myocardial edema. Circ. Res. 92 (6), 592–594. 10.1161/01.RES.0000065917.53950.75 12637366

[B157] Vanhoutte P. M. Rubanyi G. M. Miller V. M. Houston D. S. (1986). Modulation of vascular smooth muscle contraction by the endothelium. Annu. Rev. Physiol. 48, 307–320. 10.1146/annurev.ph.48.030186.001515 2871807

[B158] Vanhoutte P. M. Zhao Y. Xu A. Leung S. W. (2016). Thirty Years of Saying NO: Sources, Fate, Actions, and Misfortunes of the Endothelium-Derived Vasodilator Mediator. Circ. Res. 119 (2), 375–396. 10.1161/CIRCRESAHA.116.306531 27390338

[B159] Veresh Z. Racz A. Lotz G. Koller A. (2008). ADMA impairs nitric oxide-mediated arteriolar function due to increased superoxide production by angiotensin II-NAD(P)H oxidase pathway. Hypertension 52 (5), 960–966. 10.1161/HYPERTENSIONAHA.108.116731 18838625

[B160] Veresh Z. Debreczeni B. Hamar J. Kaminski P. M. Wolin M. S. Koller A. (2012). Asymmetric dimethylarginine reduces nitric oxide donor-mediated dilation of arterioles by activating the vascular renin-angiotensin system and reactive oxygen species. J. Vasc. Res. 49 (4), 363–372. 10.1159/000337485 22652896

[B161] Vida G. Sulyok E. Lakatos O. Ertl T. Martens-Lobenhoffer J. Bode-Boger S. M. (2009). Plasma levels of asymmetric dimethylarginine in premature neonates: its possible involvement in developmental programming of chronic diseases. Acta Paediatr. 98 (3), 437–441. 10.1111/j.1651-2227.2008.01115.x 19006524

[B162] Walker H. A. McGing E. Fisher I. Boger R. H. Bode-Boger S. M. Jackson G. (2001). Endothelium-dependent vasodilation is independent of the plasma L-arginine/ADMA ratio in men with stable angina: lack of effect of oral L-arginine on endothelial function, oxidative stress and exercise performance. J. Am. Coll. Cardiol. 38 (2), 499–505. 10.1016/s0735-1097(01)01380-8 11499744

[B163] Walter R. Mark M. Reinhart W. H. (2000). Pharmacological concentrations of arginine influence human whole blood viscosity independent of nitric oxide synthase activity in vitro. Biochem. Biophys. Res. Commun. 269 (3), 687–691. 10.1006/bbrc.2000.2344 10720477

[B164] Webb G. D. Lim L. H. Oh V. M. El Oakley R. Lee C. N. Wong P. S. (2006). Expression of neuronal nitric oxide synthase in the internal thoracic artery and saphenous vein. J. Thorac. Cardiovasc. Surg. 132 (5), 1131–1136. 10.1016/j.jtcvs.2006.08.001 17059934

[B165] Willeit P. Freitag D. F. Laukkanen J. A. Chowdhury S. Gobin R. Mayr M. (2015). Asymmetric dimethylarginine and cardiovascular risk: systematic review and meta-analysis of 22 prospective studies. J. Am. Heart Assoc. 4 (6), e001833. 10.1161/JAHA.115.001833 26021436 PMC4599532

[B166] Xuan C. Tian Q. W. Li H. Zhang B. B. He G. W. Lun L. M. (2016). Levels of asymmetric dimethylarginine (ADMA), an endogenous nitric oxide synthase inhibitor, and risk of coronary artery disease: A meta-analysis based on 4713 participants. Eur. J. Prev. Cardiol. 23 (5), 502–510. 10.1177/2047487315586094 25956428

[B167] Yang H. M. Khush K. Luikart H. Okada K. Lim H. S. Kobayashi Y. (2016). Invasive Assessment of Coronary Physiology Predicts Late Mortality After Heart Transplantation. Circulation 133 (20), 1945–1950. 10.1161/CIRCULATIONAHA.115.018741 27143679

[B168] Yuan J. Wang X. Xie Y. Wang Y. Dong L. Li H. (2017). Circulating asymmetric dimethylarginine and the risk of preeclampsia: a meta-analysis based on 1338 participants. Oncotarget 8 (27), 43944–43952. 10.18632/oncotarget.16543 28380456 PMC5546452

